# Recent trends and impact of localized surface plasmon resonance (LSPR) and surface-enhanced Raman spectroscopy (SERS) in modern analysis

**DOI:** 10.1016/j.jpha.2024.02.013

**Published:** 2024-02-28

**Authors:** Bibhu Prasad Nanda, Priyanka Rani, Priyanka Paul, Aman, Subrahmanya S. Ganti, Rohit Bhatia

**Affiliations:** a Department of Pharmaceutical Analysis, ISF College of Pharmacy Moga, 142001, Punjab, India; b Department of Pharmaceutical Chemistry, ISF College of Pharmacy Moga, 142001, Punjab, India

**Keywords:** Localized surface plasmon resonance, Surface-enhanced Raman spectroscopy, Nanophotonic, Biosensors, Nanoparticles, Biomarker, Cancer

## Abstract

An optical biosensor is a specialized analytical device that utilizes the principles of optics and light in bimolecular processes. Localized surface plasmon resonance (LSPR) is a phenomenon in the realm of nanophotonics that occurs when metallic nanoparticles (NPs) or nanostructures interact with incident light. Conversely, surface-enhanced Raman spectroscopy (SERS) is an influential analytical technique based on Raman scattering, wherein it amplifies the Raman signals of molecules when they are situated near specific and specially designed nanostructures. A detailed exploration of the recent ground-breaking developments in optical biosensors employing LSPR and SERS technologies has been thoroughly discussed along with their underlying principles and the working mechanisms. A biosensor chip has been created, featuring a high-density deposition of gold nanoparticles (AuNPs) under varying ligand concentration and reaction duration on the substrate. An ordinary description, along with a visual illustration, has been thoroughly provided for concepts such as a sensogram, refractive index shift, surface plasmon resonance (SPR), and the evanescent field, Rayleigh scattering, Raman scattering, as well as the electromagnetic enhancement and chemical enhancement. LSPR and SERS both have advantages and disadvantages, but widely used SERS has some advantages over LSPR, like chemical specificity, high sensitivity, multiplexing, and versatility in different fields. This review confirms and elucidates the significance of different disease biomarker identification. LSPR and SERS both play a vital role in the detection of various types of cancer, such as cervical cancer, ovarian cancer, endometrial cancer, prostate cancer, colorectal cancer, and brain tumors. This proposed optical biosensor offers potential applications for early diagnosis and monitoring of viral disease, bacterial infectious diseases, fungal diseases, diabetes, and cardiac disease biosensing. LSPR and SERS provide a new direction for environmental monitoring, food safety, refining impurities from water samples, and lead detection. The understanding of these biosensors is still limited and challenging.

## Highlights

• Optical biosensors offer crucial insights into molecular interactions with their precise and sensitive detection capabilities.
• We discussed plasmonic mode hybridization and multiresonant features in SERS enhance its sensitivity, contributing to advanced bio-plasmonics.
• Recent progress in designing plasmonic hotspots for SERS involves a comparative analysis of fabrication techniques.
• Exploring broad applications of LSPR and SERS, with ongoing advancements, challenges, and promising future prospects.

## Graphical abstract

**Figure:**
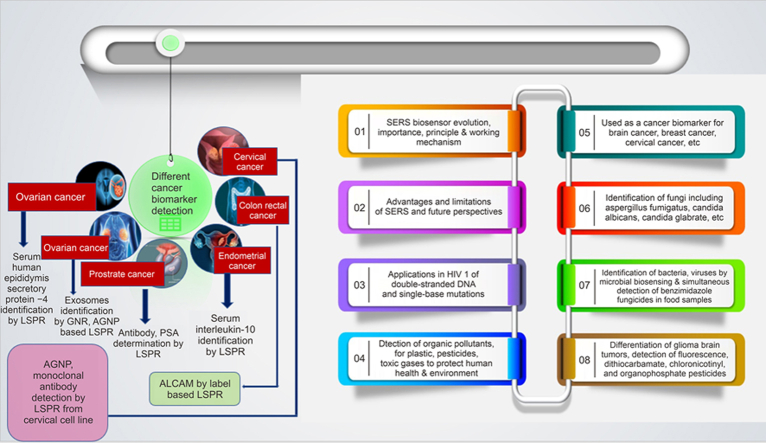

## 1 Introduction

Whether quick and easy examinations or complicated microarrays, time-resolved binding kinetics investigations, biosensors are now essential in many aspects of research, agricultural production, and healthcare [1,2]. Creating detection tools and testing their sensing capabilities are both necessary for the development of biosensors. A substrate and receptor are commonly chosen during fabrication, and afterward, the substrate and receptor are functionalized [3]. Different kinds of biosensors are available in the constantly expanding market for a range of applications [1,4]. The goal of biosensors [5] is generally to measure the signal intensity and accuracy, ensuring that they meet the desired sensitivity, specificity and effectiveness requirements. Biosensors can indeed be categorized based on the type of transducer they use to convert the interaction between the bioreceptor and the analyte into a measured signal. However, it’s worth noting that the field of biosensors is continually evolving day by day, and the hybrid biosensor concept [6] is a burning topic all over the world. Optical biosensors and thermal biosensors [7] are the two primary categories of biosensors based on transduction techniques, and other types of biosensors include optical biosensors, thermal biosensors [7,8], piezoelectric biosensors [9], quartz crystal microbalance biosensor [10], and electrochemical biosensors [[11], [12]].

Optical biosensors, particularly plasmonic ones, offer several benefits that make them very useful for the detection and monitoring of a variety of substances and biomolecules [13]. Generally, plasmonic sensors provide high sensitivity and multiplexing capabilities [14]. Localized surface plasmon resonance (LSPR) spectroscopy is gaining popularity due to its reasonable cost, high sensitivity, and label-free detection [3,15,16]. Surface plasmon resonance (SPR) [17,18], surface-enhanced Raman scattering (SERS) [19], long-range surface plasmon polariton (LRSPP) [20] and many forms of plasmonic sensing devices are widely used in the modern age. These include several types of plasmonic sensors, such as LSPR, which is localized surface plasmon resonance [21,22].

**Fig. 1:**
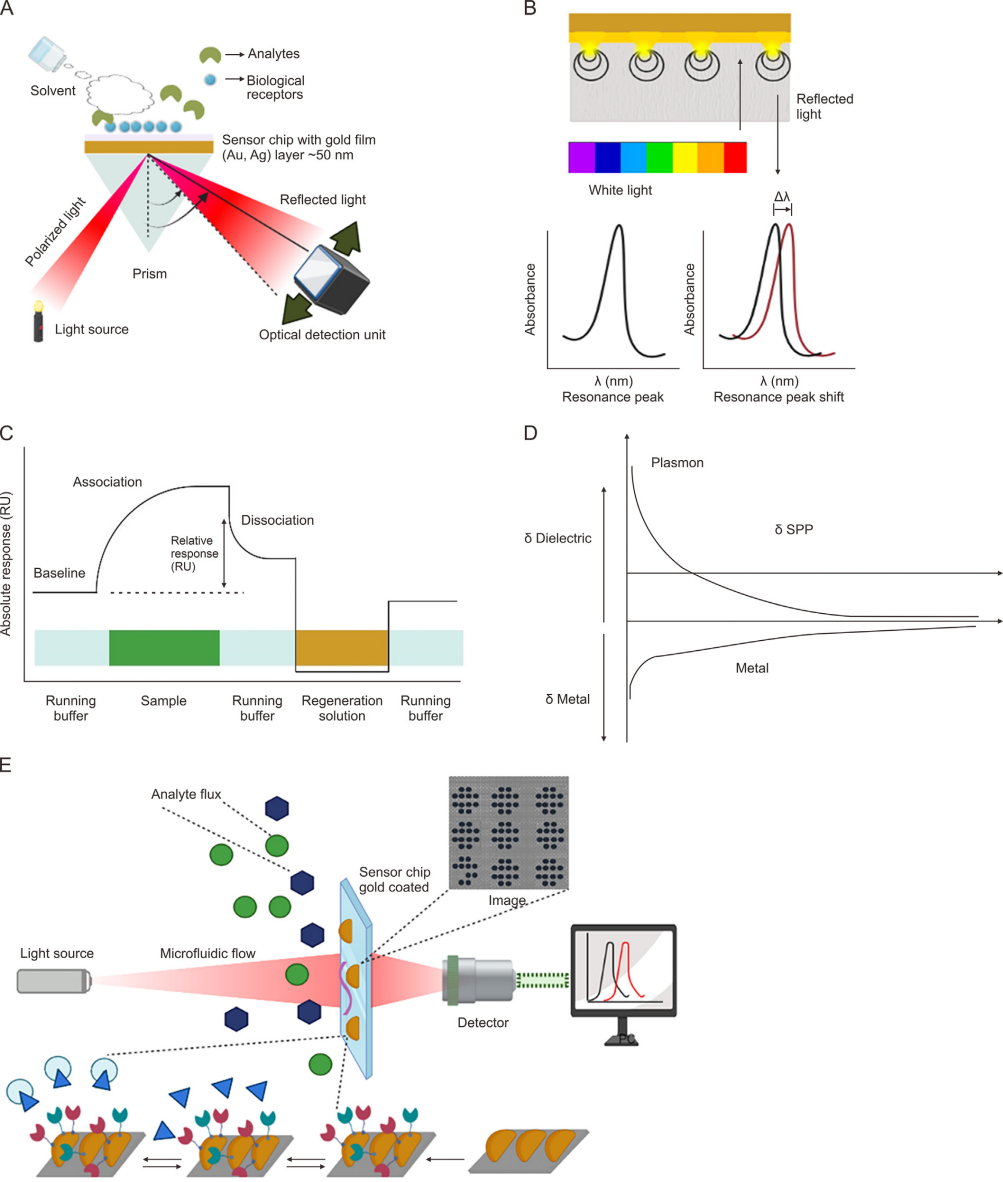
Basic localized surface plasmon resonance (LSPR), surface plasmon resonance (SPR) principles, working & concept of sensogram. (A) The schematic diagram of conventional SPR set up with prism. (B) Flexible LSPR technology with refractive index (RI) shifted graphical representation. (C) A typical explanation of sensogram of changing RI due to specific binding. (D) δ dielectric-evanescent field of the dielectric, δ metal – evanescent field in the metal & δ surface plasmon polarization (SPP). (E) Schematic representation of strongly confined LSPR-based sensor using transmission attenuation.

Light waves trapped inside conductive nanoparticles (NPs) [23] that are smaller than the wavelength of light cause the optical phenomenon known as LSPR through the interaction of surface electrons in a conduction band with incoming light. The gold nanoislands (AuNIs) chip in the biosensor is functionalized with a complementary DNA probe to detect the RNA sequence [24] through the hybridization of nucleic acids [25]. The interactions are monitored on a removable chip by an SPR detector. Different matrices can be used for interactions depending on the nature of the molecules being immobilized [3,15]. The wedge-shaped beam of polarized light focuses through a prism into the sensor chip [26] and the SPR response is monitored by the detector [27] as explained in Fig. 1A. The angle at which the mean light reflection occurs is called the resonant angle, which is related to the refractive index (RI and mass of the molecules attached to the sensor chip. When the surface plasmon is localized, the lateral diameter of the interface is less than the evanescent field of surface plasmon propagation, as well explained in Fig. 1B, which shows that a surface plasmon polariton (or propagating plasmon) and a localized surface plasmon provide a fundamental understanding of how plasmons are influenced by local structure and environment, and they also suggest the usefulness of plasmons as a sensing modality [15]. When a beam of light moves from air or water, which are non-conductive materials commonly referred to as dielectrics, and encounter the boundary between the metal and the dielectric material, something extraordinary occurs. The wave travelling along the interface at the impinging point of light is called a surface plasmon polarization (SPP) [28]. An SPR sensogram is a plot of SPR response vs. time generated by an SPR instrument, which provides information related to the binding event between an analyte (e.g., antibody) and a ligand (e.g., protein). It also gives pieces of information about the specificity of binding. The sensogram [29] helps to know the kinetic, affinity and concentration information of the analytes and ligands. The RI calculated by the ligand attached to the matrix serves as a baseline measure, as discussed in Fig. 1C. The analyte or other ligands are injected in a buffer or aqueous solution over the chip and either bind or get washed back out into the waste bottle. As the analyte binds to SPR angle changes, the RI is caused to change. Graph of change in RI due to binding interactions is presented in Fig. 1C. Once the ligand is immobilized, a baseline response is collected, and the response decreases when the buffer solution is injected, causing two molecules to dissociate after the completion of analyte bindings [30]. A regeneration solution is injected to remove the analyte and provide a clean slate for the next analyte, which is shown in Fig. 1C. Fig. 1D demonstrates δ dielectric-evanescent field of the dielectric, indicating how far surface plasmon wave extends [31]. δ metal refers to an evanescent field in the metal, and δ SPP refers to surface plasmon propagation (how far from the excitation point the surface plasmon can travel along the metal or dielectric interface).

With an excitation light with a wavelength of around 500 nm, the characteristics of the resulting plasmon lengths in SPR were examined using a planar gold surface.

The plasmon field in metal is in the order of a few nanometers, indicating that the surface plasmon does not penetrate deeply into the metal.

If the lateral dimension of the interface ≪ δ SPP, surface plasmon is localized.

δ Dielectric 20–40 nm for LSPR [31]

Light interacts with particles that are considerably smaller than the incoming wavelength due to localized surface plasmons (Fig. 1D). As a result, a plasmon with the LSPR frequency starts to oscillate around the NP. The LSPR is sensitive to changes in the local dielectric environment, much like the SPR. In Fig. 1E, it is visible that analyte flux is applied to the gold-coated sensor chip, with the microfluidic flow and the laser beam applied to the chip. The interaction between the ligand and proper receptor occurs, and then the LSPR wavelength becomes high. The high-quality image is recorded by atomic absorption microscopy and is easily detected by the detector. Scattering of light by the molecule can be elastic for Rayleigh scattering [32], or maybe inelastic, famously known as Raman scattering [16]. Possibilities of SERS as a potent chemical sensing platform and an emission method that uses inelastic scattering, where the frequency of the monochromatic light changes upon interaction with vibrational stages or modes of a molecule. It is one of the highly sensitive techniques used to probe structural details of a complex molecule. In SERS, methods molecules adsorbed on the gold/silver surfaces with rough surfaces increase the Raman scattering [21,22]. In SERS, When incident light is absorbed by the molecule, it leads to excitation to a virtual state, resulting in a red shift and providing a molecular fingerprint. It is a rare and challenging phenomenon, but it has been known for a long time and has numerous applications in the biomedical field [16].

For background scattering-based internal standards for plasmonics SERS biosensing calibration, the calculation of enhancement factor (EF) is performed. Accurately calculating EF values is difficult due to the need for model analytes and assumptions about the number of analyte molecules in the laser excitation area. Wei et al. [33] was the first to propose an alternative evaluation parameter for SERS substrate performance based on the intensity of the surface plasmon-enhanced Rayleigh band, which originates from the amplified spontaneous emission (ASE) of the laser. They utilized six varied SERS substrates, including different states (solid, suspended in liquid, and hydrogel), distinct plasmonic NP types (silver and gold), and diverse sizes and shapes of NPs, to validate their hypothesis. The results show significant relationships between I_Rayleigh_ and measured SERS intensities, as well as between SERS uniformity and I_Rayleigh_ variations across the six distinct SERS substrates.

**Fig. 2:**
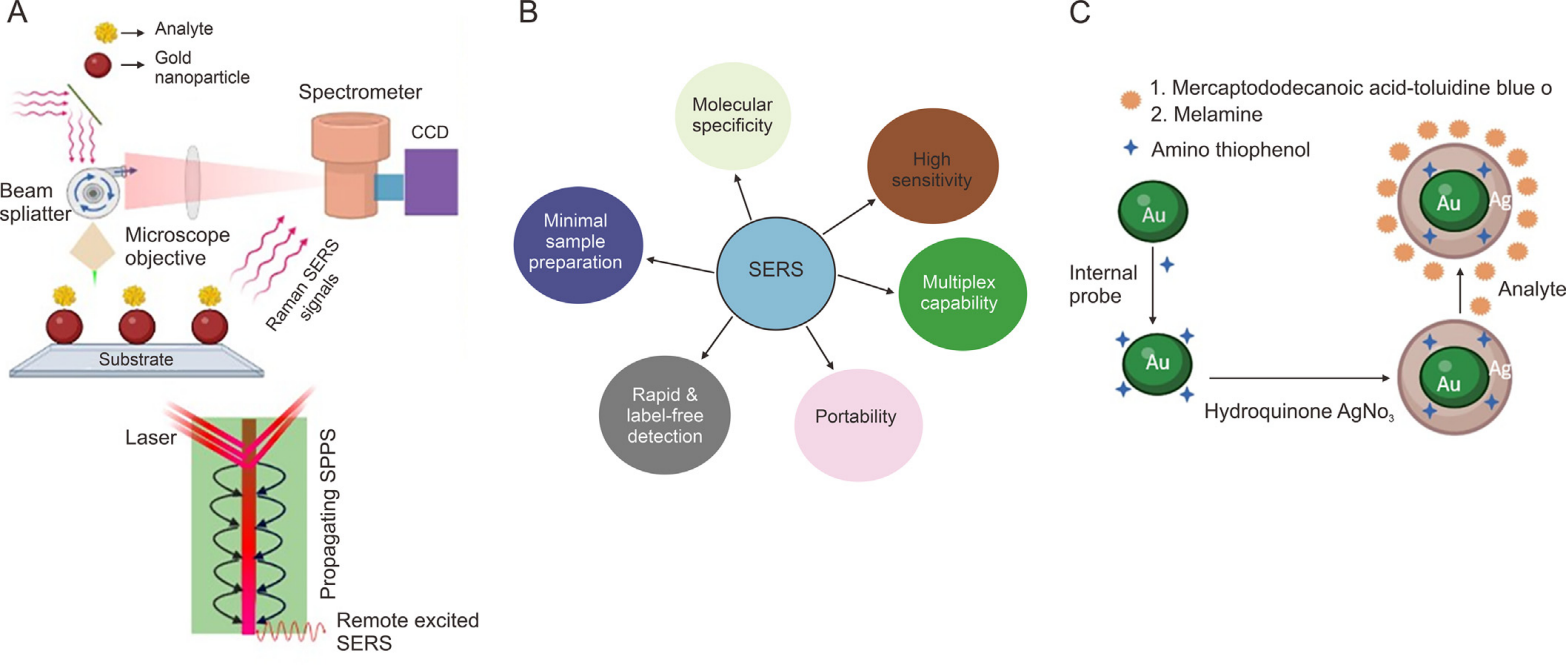
Demonstration of charge transfer effect and advantages of surface-enhanced Raman spectroscopy (SERS). (A) Schematic comparative visualization of the charge transfer effect of SERS. (B) The advantages of SERS biosensors over localized surface plasmon resonance (LSPR) biosensors. (C) A diagram representing the quantitative SERS measurement using Au-core/Ag-shell nanoparticles with an incorporated internal reference. CCD: central composite design.

Theoretical and experimental analysis indicates that light, amplified by stimulated emission, which is elastically scattered by a specific region known as a SERS “hotspot”, accurately reflects the overall strength of the localized electromagnetic field. The number of target analytes (NA) within a “hotspot” determines the ratio between the elastic and inelastic scattering signals, regardless of variations in the size, shape, pattern, and density of plasmonic nanostructures. The use of SERS for routine quantitative analysis is limited by the variability in SERS substrates from one point to another. This variability occurs because of the uneven distribution of localized electromagnetic fields across different plasmonic nanostructures. Wei et al. [34] adopted surface-enhanced elastic scattering as a SERS internal standard. A localized intrinsic internal standard that scales over all of the plasmon-enhanced electromagnetic fields within a substrate is provided by the surface-enhanced elastic scattering signal. The light that is elastically scattered originates from the ASE of the commercial laser. This process results in the creation of a pseudo band with low wavenumbers, which occurs due to the interaction between ASE and the edge filter. This approach offers highly consistent data on analyte adsorption, in both static and dynamic imaging scenarios. It significantly improves the quantification of four chloroanilines using SERS. Overall, the SERS [35] system amplifies Raman signals using the special qualities of metal nanostructures, making it possible to analyze molecules with extreme sensitivity and specificity even at low concentrations. This method has several applications in a variety of disciplines, including forensic investigation, biology, medicine, environmental monitoring, and materials research. In Fig. 2A, the process begins with the laser source that emits an intensified beam of light. The SERS substrate in rough surface metals of gold particles and the laser collides with analytes, and enhanced Raman SERS signals are recorded by the spectrometer. When the laser propagation of SPP [36] occurs, remote excited SERS signals are also observed. Two common mechanisms could be used to explain SERS enhancement in most systems based on different nanomaterials: (a) electromagnetic enhancement due to LSPR [37,38] where oscillation of electrons occurs at the surface of the metals as a result of an enhanced electric field, leading to changes in the optical properties of the molecule, as a result Raman scattering occurs [39]; and (b) chemical enhancement [40], which involves charge transfer between the adsorbed molecule and the metals and non-metals substrates, depending on the electronic states of the SERS substrate, when a molecule is located on the surface of a semiconductor (metal oxides) or carbon nanomaterials [16,41,42]. In sensor technology, nanomaterials have become increasingly popular in recent decades. It is believed that adding materials at the nanoscale can positively improve sensor performance. Nanomaterials have the potential to completely transform sensors because of their numerous interesting and unique physical and chemical properties. LSPR offers several inherent advantages over SPR [43], and these advantages have contributed to widespread uses such as biosensing, environmental monitoring, and material characterization [44]. LSPR offers label-free detection for several bioassays and protein microarrays [45], and tracks the entire course of cell signaling [46] in real time. LSPR plays a vital role in observation under room light and human eyes, and even in some cases, changes in the LSPR signals can be easily observed by the naked eyes, providing an easy and immediate way of detecting reactions. LSPR is so famous for its simple and compact instrumentation. This technique has very minimal bulk effects. Now in the modern era, affordable sensor chips of LSPR provide more economical options in the biosensing field. LSPR is widely used because strict temperature control is not required, which reduces the need for accurate temperature control. Like other techniques, LSPR is not critically dependent on the angle of incidence of light, minimizing the impace of mechanical noise and vibrations. Thus, LSPR can be considered as an eco-friendly, user-friendly, and a straightforward technique [47,48]. Highly sensitive SERS encounters challenges in quantitative analysis due to its sensitivity to fluctuations in local optical fields at plasmonic hotspots within metallo-dielectric nanostructures. Raman tag-based current SERS calibration techniques are limited by spectrum interference and spatial occupancy competition with analyte molecules with photodegradation and analyte Raman peaks. Sharma et al. [49] reported that plasmon-enhanced electronic Raman scattering (ERS) signals from metal can serve as an internal standard for spatial and temporal calibration of molecular Raman scattering (MRS) signals from analyte molecules at the same hotspots, enabling rigorous quantitative SERS analysis. They found a direct relationship between the signal intensities of ERS and MRS when there were changes in the excitation optical fields, both spatially and temporally. This finding demonstrated |E|^4^ enhancements for both ERS and MRS processes occurring at the same hotspots, aligning with our theoretical predictions. Some basic advantages of SERS over LSPR have been described in Fig. 2B.

SERS can be performed with high sensitivity with more traditional methods like fluorescence. By using SERS, we can get fingerprints that allow us to distinguish multiple different molecules at the same time. As the technology has improved, these Raman spectroscopies have become increasingly portable [50,51].

## 2 Basics of plasmonic mode hybridization and multi-resonant plasmonic characteristics in SERS

Prodan et al. [52] introduced a straightforward and clear concept, which serves as an electromagnetic analogy to molecular orbital theory. This concept helps explain the plasmon behavior in intricate nanostructures of various shapes. Their model can be considered as the interaction or “hybridization” of basic plasmons that are supported by nanostructures with elementary geometries. The study focused on plasmon resonances in hollow metallic nanospheres, or nanoshells. The inner and outer radii of these nanoshells have a significant impact on their plasmon frequencies. They created an energy-level diagram that showed how the interaction between cavity plasmons and sphere plasmons causes plasmon hybridization in metal nanoshells. The radius of the core defined the concentric nanoshell geometry. Comprehensive quantum mechanical computations were performed to verify the veracity of their statement regarding nanoshell plasmon energies. The method was employed to analyze a significant scenario involving a four-layer concentric nanoshell. In this case, the resonant frequencies of the multilayer nanostructure are determined by the interaction or hybridization of plasmons in the inner and outer nanoshells.

Tali et al. [53] provided an overview of multi-resonant plasmonics with spatial mode overlapping. They explained that plasmonic nanostructures have characteristics of photodetection, light emission, optical biosensing, and spectroscopy. Traditional plasmonic devices and systems are usually designed to work effectively within a specific wavelength range, which limits their suitability for multiband nanophotonic applications. These applications may either require enhanced nanoplasmonic effects for multiphoton processes in quantum systems at multiple resonant wavelengths or demand wavelength-multiplexed operations at the nanoscale. The researchers developed techniques to achieve "multitenant plasmonic" systems, which enable enhanced light-matter interactions across multiple wavelength bands at the same locations in nanoplasmonic systems. They provided an overview of recent progress in the research of multi-resonant plasmonic systems featuring spatial mode overlap. Specifically, they discussed and highlighted the technique of “plasmonic mode hybridization” as a universal approach for designing and constructing multi-resonant plasmonic systems with overlapping spatial modes. They discussed the unique and significant role that spatial mode overlap in multi-resonant plasmonics can play in a range of existing and future applications. These applications include optical nanodevices with multiple modes and bands that enable wavelength multiplexed optical multimodalities on a nanoscale scale, as well as nanodevices for multiphoton nonlinear optical and upconversion luminescence, which benefit from simultaneous enhancement of optical excitation and radiation processes across multiple wavelengths.

## 3 Plasmonic modes with their pros and cons for bio-plasmonics

Khan et al. [54] discussed tunable sub-radiant lattice plasmons through out-of-plane dipolar interactions. The short duration of emissive plasmons limits their energy enhancement potential due to rapid depletion, which hinders further enhancement of local optical fields. Creating nanostructures at scale is challenging due to their complex, subwavelength shapes. However, a new sub-radiant plasmon with a narrow resonant linewidth of about 5 nm has been introduced. This plasmon can be easily tuned by adjusting the height of large gold nanoparticles (AuNPs), exceeding 100 nm, arranged in a two-dimensional (2D) array. Close interaction between the vertical dipolar moments of the NPs prevents radiative decay during resonance, trapping light within the array plane and intensely focusing optical fields on specific NPs. This innovative process opens the possibility of creating height-controlled NP arrays on large wafers made of various substrates. When the extinction peak at 750 nm is analyzed, it reveals a narrow dip (FWHM ≈ 10 nm) at 758 nm within a broad peak (FWHM ≈ 100 nm) when decomposed into its scattering and absorption components.

Zhou et al. [55] studied delocalized lattice plasmon resonances showing dispersive quality factors. The out-of-plane lattice plasmon resonances in 2D arrays of AuNPs exhibit dispersive quality factors, which can be customized by adjusting the height of the AuNPs. Computational analyses were carried out to study the near-field optical properties and band diagrams, helping to comprehend the observed dispersion effects of the out-of-plane lattice plasmons. The findings showed that the delocalized out-of-plane lattice plasmons (OLPs) are a surface Bloch mode composed of multiple Bloch harmonics. As the OLP dispersion changes from a stationary state to a propagating state, there is a decrease in nonradiative loss due to limited local field confinement, while radiative loss increases because of intense coupling with the leaky zero-order harmonic. To further investigate the dispersion effects observed in OLP resonances, three-dimensional finite-difference time-domain (FDTD) simulations were performed. The goal of these computations was to derive the 2D periodic arrays of AuNPs and their near-field optical characteristics. Suh et al. focused on the hybridization of localized and guided modes in 2D metal–insulator–metal (MIM) nanocavity arrays. They studied how the optical characteristics of 2D MIM nanocavity arrays change with different angles. Using a combination of soft nanolithography and template stripping techniques, they created arrays of plasmonic MIM nanostructures with subwavelength spacings, covering square centimeter areas. They managed to adjust the interaction between localized surface plasmons and guided modes by modifying the thickness of the insulator, thereby manipulating the optical band structure. The extent of Rabi splitting in hybridized modes was significantly influenced by how closely the near-fields of localized and guided modes overlapped.

Tali et al. [56] reported that to achieve concentrated nanoscale light across a wide range of wavelengths, a viable approach involves using plasmonic devices with multiple resonances. These devices support various hybridized surface plasmon modes, all of which overlap spatially at different resonance wavelengths. Plasmonic modes resulting from hybridization at higher orders often exhibit a dark multipolar characteristic and are less effective due to weak interactions with light in free space. Studies have shown that plasmonic crystals arranged in a two-tiered nanolaminate arrangement can support up to 10 hybridized plasmonic modes. These modes are very sensitive and spatially overlapped, especially when exposed to free-space light with wavelengths ranging from 400 to 1,400 nm. Massive plasmonic crystals, or nanolaminates, can be produced by combining multilayered physical vapor deposition techniques with nanoimprinting lithography. The tightly spaced subsystems that comprise these crystals include nanolaminate, nanodome, and nanohole arrays. The multi-resonant plasmonic responses of the crystals are confirmed by the excellent agreement between experimental observations and theoretical computations. Several spatially overlapped and highly excitable hybridized plasmonic modes may be supported by the nanolaminate plasmonic crystals in a two-tier structure, enabling multi-resonant nanoscale light concentration throughout a broad wavelength range of 400–1,400 nm. They envisioned that multi-resonant nano-optics platforms, such as nanolaminate plasmonic crystals, could be used to boost concurrently numerous excitation/emission transitions of multiphoton nonlinear optical processes across a broad wavelength range.

## 4 Molecular Raman tag-based internal standards for plasmonics SERS biosensing calibration

Lorén et al. [57] presented the use of self-assembled monolayers (SAMs) as internal standards for calibration in SERS. Three compounds containing cyano groups were linked to gold colloids through a metal–sulfur bond, and their spectral stability and normalization capacity were examined. SERS were obtained by illuminating the samples with a 633-nm He/Ne laser using a 60× water-immersion objective with a numerical aperture of 1.3. The scattered light was filtered, dispersed and recorded with a cooled near-infrared (NIR) enhanced central composite design (CCD) using a Labram INV micro spectrometer from Jobin-Yvon. XY maps were generated by scanning over the metal clusters with a 1-μm step size. Principal component analysis (PCA) and partial least squares (PLS) were performed in Simca 10.0.4 (Umetrics AB). The theoretical behavior of a system containing the analyte and internal standard was simulated through the multivariate analysis. The findings indicated a simultaneous increase in signal enhancement for both the analyte, rhodamine 6G, and the internal standard. This co-variation allowed for the quantification of the analyte via partial least squares (PLS) analysis. Chen et al. [58] discussed large-scale hotspot engineering for quantitative SERS at the single-molecule scale. SERS requires precise control of the SERS substrate’s detection uniformity and Raman EF. The study demonstrates that silver nanoparticle (AgNP) films regulated by alkane thiolate ligands can enable precise quantitative SERS measurements, even at the level of individual molecules. These films’ 2D hexagonal close-packed AgNP superlattices provide outstanding homogeneity and a high Raman EF for SERS detection across a wide region. They proved the effectiveness of quantitative SERS by determining the surface densities of crystal violet molecules within a highly enhanced region called a “hot zone.” This zone is planar, uniformly intensified, and located a few nanometers above the AgNPs. The Raman measurements showed a consistent response across a broad range of analyte concentrations, indicating a linear relationship.

Shen et al. [59] performed a reliable quantitative SERS analysis facilitated by core-shell NPs with embedded internal standards. Core-molecule-shell NPs were rationally designed for quantitative SERS analysis, with two components in the molecular layer, a framework molecule to form the shell, and a probe molecule as a Raman internal standard. The signal from the Raman probe integrated within the sample offers valuable feedback, enabling the correction of fluctuations in samples and measurement conditions. Additionally, molecules with varying affinities can be adsorbed onto the outer shell. Conventional SERS methods have not been able to perform the quantitative analysis of target molecules over a vast concentration range with a linear response of the relative SERS intensity with the surface coverage. A new quantitative SERS technique was developed utilizing core-molecule-shell NPs. The molecular layer consists of two components with distinct functions: one assists in shell formation, while the other produces a robust Raman signal and serves as the internal standard.

Zhou et al. [60] developed quantitative surface-enhanced Raman measurements with an embedded internal reference. SERS is frequently used for qualitative rather than quantitative analysis in analytical applications due to the challenges in achieving accurate quantitative results. In this study, a new strategy was introduced to quantitatively measure the SERS signals of analytes based on Au-core/Ag-shell NPs with embedded 4-amino thiophenol as the internal reference. The study successfully identified two substances, toluidine blue O in water (detectable at 0.1 mM) and melamine in milk (detectable at 5 mM), which is shown in Fig. 2C. By employing an internal reference, the accuracy of quantitative SERS measurements was significantly improved, as indicated by enhanced linear fitting. The ability to detect melamine in milk highlights the adaptability of this detection method for various analytes. The design of the Au-core/Ag-shell NP with embedded internal reference is illustrated in Fig. 2C. The method for synthesizing internal reference-embedded Au-core/Ag-shell NPs is similar to previous reports, with minor modifications.

## 5 Recent advancements in engineering plasmonic hotspots for efficient bio-nanoplasmonics and SERS devices, a comparative analysis of the different fabrication methods

### 5.1 In-plane plasmonic nanogap hotspots in plasmonic biosensors

Chen et al. [61] described atomic layer lithography of wafer-scale nanogap arrays for extreme confinement of electromagnetic waves. Passing light through tiny gaps in metals that are just a few nanometers wide can lead to significant increases in field strength, nonlocal electromagnetic phenomena, and electron tunneling triggered by light. They developed a novel patterning technique using atomic layer deposition and straightforward adhesive-tape-based smoothing. The method enabled the creation of vertically oriented gaps in solid metal films acrossing a millimeter-sized pattern, with the gaps as narrow as 9.9 Å. They managed to fit 150,000 of these devices on a 4-inch wafer. The nanogaps allowed electromagnetic waves to pass through without interference from the background, enabling clear transmission measurements. The researchers observed resonant transmission of NIR waves through incredibly narrow 1.1-nm-wide gaps (1/1295) and calculated an effective RI of 17.8. Additionally, they observed the resonant transmission of millimeter waves through extremely narrow 1.1-nm-wide gaps (1/4,000,000) and deduced an extraordinary field EF of 25,000, a phenomenon that has never been observed before.

Hu et al. [62] discussed gold nanofingers for molecule trapping and detection. They presented a molecular trap design capable of capturing specific molecules in a solution, facilitating their detection and identification. This design employs nanoscale polymer fingers coated with gold, fabricated through a nanoimprinting method. These flexible nanofingers can be manipulated to capture molecules, with the gold-coated tips serving as a reliable Raman hotspot. This configuration enables molecule detection and identification via SERS. The controlled gap size between the fingertips, controlled by the molecules themselves, guarantees maximum enhancement for sensitive molecule detection using SERS. Additionally, structures created by combining top-down and self-assembly methods can be applied to various fields like plasmonic, metamaterials, and other nanophotonic systems.

Fu et al. [63] introduced highly reproducible and sensitive SERS substrates with Ag inter-NP gaps of 5 nm, fabricated using an ultrathin aluminum mask technique. They described an easy and affordable manufacturing method for producing a large-scale SERS substrate. This substrate with silver inter-NP gaps measuring 5 nm, was achieved through the use of an ultrathin alumina mask (UTAM) surface patterning technique. Densely packed arrays of AgNPs with strong electromagnetic field enhancement points (“hotspots”) over a large area demonstrate high SERS activity and consistent reproducibility. The SERS performance was evaluated using Rhodamine 6G molecules at a concentration of 1 × 10^−7^ M, resulting in an EF of approximately 10^9^. They achieved highly consistent signals, with a relative standard deviation (RSD) of around 2% from 10 randomly selected spots within a 1 cm area. This level of reproducibility is significant. The enhanced electric field results from the narrow gap, aligning with our experimental outcomes. The combination of low RSD values and the high EF in SERS signals suggests that the prepared substrate holds great promise for uniform and highly sensitive SERS detection. It is possible to realize nanopatterns with even smaller spacing than 5 nm via patterning process, leading to higher SERS EF.

Zhang et al. [64] proposed the concept of hierarchical porous plasmonic metamaterials for reproducible ultrasensitive SERS. They explained that a multiscale architecture not only facilitates efficient cascaded electromagnetic enhancement but also provides an enormous number of Raman-active binding sites, exhibiting excellent reproducibility and ultrasensitive detection of aromatic molecules down to 10^−13^ M.

The typical explanation of fabrication and characterization of hierarchically ordered porous metamaterials, preparation of the negative poly (methyl methacrylate) (PMMA) template from porous anodic alumina, and removal of the PMMA template has been explained. Ensuring consistent SERS reproducibility is just as crucial as achieving high SERS sensitivity. This reliability is essential in designing SERS-based sensors, enabling the accurate quantification of specific analytes.

Garg et al. [65] developed reusable SERS membranes and textiles through template-assisted self-assembly and micro/nanoimprinting. The creation of wearable SERS devices involved applying plasmonic NPs directly onto fabrics, but this method resulted in uneven NP assembly, with particles weakly adhering to the fabric due to van der Waals forces. They described the development of reusable SERS membranes and textiles that can be washed. This was achieved through template-assisted self-assembly and micro/nanoimprinting techniques. They used a capillary force-driven self-assembly method to create arrays of AuNP aggregates within microstructured templates with hydrophobic properties. These aggregates were then securely attached to semipermeable transparent membranes and stretchable textiles using a micro/nanoimprinting technique based on ultraviolet (UV)-resistant materials. For even greater SERS performance, textiles and SERS membranes treated with light reactive ion etching (RIE) can physically reveal the AuNP-aggregate SERS hotspots contained in the polymer UV resist. They showed that semipermeable transparent SERS membranes prevent the moisture in meat from evaporating, enabling stable *in situ* SERS monitoring of biochemical environments on fresh meat surfaces. In contrast, stretchable SERS textiles allow the spreading, soaking, and evaporation of analyte samples on the fabric, enriching analyte molecules at hotspots for continuous biochemical SERS detection. Their conclusion highlighted that washing reusable SERS membranes and textiles through template-assisted self-assembly and micro/nanoimprinting techniques present a great potential for wearable biochemical sensing applications. These applications include areas like wound monitoring and body fluid analysis.

### 5.2 Out-of-plane plasmonic nanogap hotspots in plasmonics biosensors

Song et al. [66] developed scalable, high-performance nanolaminated SERS substrates based on multistack vertically oriented plasmonic nanogaps. Metallic nanogap structures can sustain surface plasmon modes within the gaps and significantly concentrate optical fields, enabling label-free biochemical analysis using SERS at the level of individual molecules. Existing scalable SERS substrates, which rely on horizontally oriented plasmonic nanogaps, encounter difficulties in achieving precise control of in-plane nanostructures at dimensions below 10 nm. They introduced an innovative and scalable SERS substrate with superior performance. This substrate is built on vertically aligned nanogap hotspots within metal-insulator–metal nanolaminated plasmonic crystals. Unlike horizontally oriented nanogaps, vertically aligned plasmonic nanogaps can be precisely controlled at subnanometer levels during the multilayered thin-film deposition process. Through partial etching of dielectric layers, embedded nanogap hotspots within nanolaminate SERS substrates can be exposed, leading to a significant increase in SERS EFs by more than tenfold, from around 1 × 10^7^ to approximately 1.6 × 10^8^. Their research introduces an innovative method for plasmonic engineering in the out-of-plane direction, enabling the design and production of scalable, high-performance, and reusable SERS substrates. These substrates are well-suited for biochemical analysis applications that require high spatial-temporal resolution and uniform distribution of hotspots.

Nam et al. [67] explored Au/SiO_2_-nanolaminated plasmonic nanoantennas as refractive-index-insensitive and transparent SERS substrates. Because the SPR wavelength is highly influenced by the RI of the surrounding plasmonic nanostructures, the EFs in SERS hotspots are responsive to shifts in the background RI. This sensitivity presents a challenge for quantitative SERS biochemical analysis in practical scenarios where the RI varies spatially and temporally. The study presents a platform called tapered-shape nanolaminate plasmonic nanoantenna (TNLNA), which supports multiple magnetoelectric localized surface plasmon modes that overlap spatially. This configuration demonstrates remarkable SERS EFs (>10^7^) and remains unaffected by variations in background refractive index, ranging from 1.30 to 1.60. They finally demonstrated that the uniform arrays of TNLNAs can be manufactured on flexible transparent polymer films to achieve backside-excitable and reversible SERS measurements for *in situ* label-free glucose monitoring on a skin phantom.

Excellent photostability under laser stimulation, minimal spectrum interference with biomolecule Raman signals, and no occupancy competition with biomolecules at hotspots are the benefits of the ERS-based SERS calibration. Thus, it is anticipated that the multivariate analysis performance in label-free SERS measurements of living biological systems and other intricate biochemical matrices may be greatly enhanced by the ERS-based SERS calibration. NPs has a significant impact in the enhancement of LSPR and SERS signals for biosensor applications, such as increasing the sensitivity of the system, and creating repeatable hotspot locations for single-molecule SERS measurements. Nanostructures possess distinct characteristics and respond naturally to their surroundings, making them ideal candidates for biosensor design. Typically, alterations in the magnetic, optical, and/or electrical properties of these nanostructures are harnessed for sensing purposes.

## 6 Applications of LSPR

### 6.1 LSPR biosensing in cancer and cardiac disease

Zhao et al. [68] demonstrated the use of the widely used anti-cancer drug methotrexate (MTX) on the human dihydrofolate reductase enzyme (hDHFR). In a competitive binding experiment relying on LSPR, researchers utilized AuNPs modified with folic acid (FA-AuNPs). Characterization of FA-AuNPs was performed successfully, and the dynamic range of the competitive assay based on LSPR for MTX in saline solution was found, along with interference from other known ligands of hDHFR. Clinical samples with an MTX therapeutic level monitoring chart provide the information regarding comparison of the LSPR technique for MTX quantification to liquid chromatography tandem mass spectrometry (LC-MS/MS) and fluorescence polarization immunoassay (FPIA). To measure and detect MTX, a rapid analytical technique utilizing LSPR sensors has been developed. This method depends on a change in LSPR that is inversely correlated with the concentration of MTX and is brought on by the specific binding of FA-AuNPs to the free enzyme. The detection dynamic range for MTX was increased from 10^−11^ M to 10^−6^ M by altering the concentration of hDHFR from 1 to 100 nm. Clinical samples of human serum from MTX chemotherapy patients were analyzed, and a detection limit (LOD) of 155 nm was obtained using a straightforward solid-phase extraction process for the separation of MTX from the matrix of serum. A calibration curve was used in correlation tests to evaluate the quantification of MTX by LSPR, and the results showed an *R*^2^ value of 0.947. In conclusion, a quick analytical method based on LSPR sensors has been developed to detect and quantify MTX. Calibrations have been carried out in the nanomolar range in both peripheral blood smear (PBS) and fetal bovine serum, the latter of which requires the use of SPE cartridges.

Hou et al. [69] explained that gold nanorods (GNRs) with proper aspect ratios are often used for imaging purposes, both *in vitro* and *in vivo*, notably in the context of photothermal cancer treatment due to their strong LSPR in the 650–900 nm NIR range. In their work, a durable and effective photothermal agent for killing cancer cells was created by coating GNRs with biocompatible polyaniline (PANI). Surprisingly, cancer cells were efficiently destroyed with a 0.6 W/cm^2^ laser power, the lowest value yet recorded for plasmonic nanostructures. This was accomplished by continuously exposing the cells to an 808 nm NIR laser for 5 min . The considerable potential of photothermal therapy as a cancer treatment strategy is shown by these findings.

Aćimović et al. [70] introduced a parallel, quick, and sensitive cancer marker detection using an LSPR chip in serum. J. N et al. [70] introduced the first lab-on-a-chip technology that significantly outperforms current technologies by combining the most recent developments in plasmonic, nanofabrication, and microfluidics, using parallelized LSPR. With extremely high reproducibility and repeatability, their system provides simultaneous, real-time examination of 32 sensor locations dispersed throughout eight separate microfluidic channels. As a result, several biomolecule sensing techniques were evaluated. Zhang et al. [71] established NPs of Cu_2__-__–__x_Se for dual-modal imaging-guided photothermal therapy of cancer, featuring tunable LSPR and magnetism, using vacancy specifically show how a complex matrix including 50% human serum can quickly identify prostate-specific antigen and human alpha-fetoprotein, two major cancer indicators, present in amounts as low as 500 pg/mL. They found a way to control a special type of light-absorbing property in tiny structures by adding magnetic ferric ions (Fe^3^^+^). They also adjusted the gaps in another type of NP by mixing them with Fe^3^^+^ at room temperature. Computer simulations confirmed that both adding Fe3^+^ and changing the NPs’ composition are beneficial. The resulting hybrid structure has promising uses in medical imaging and cancer treatment. It can absorb specific types of NIR light and generate sound waves for imaging deep inside tissues. The advancement opens up new possibilities for innovative ways for targeted drug therapy, and is also useful for magnetic resonance imaging (MRI).

Ki et al. [72] developed enzyme-assisted target recycling system and a newly designed LSPR probe for the sensitive plasmonic detection of miR-10b in biological samples. They described an enzyme-assisted target recycling system-based LSPR and an LSPR probe to identify miR-10b, a significant miRNA for stomach cancer. Personalized medicine and cancer surveillance can both benefit greatly from this integrated detection systems approach to the sensitive detection of miRNAs, which help in cell differentiation, cell cycle progression, and function as oncogenes. The suggested sensing platform was demonstrated using microRNA-10b (miR-10b), and fluorescence analysis was used to verify that target recycling with DSN support was successful. Sensitivity and selectivity were assessed under the best circumstances. The LOD was established using sequential dilution that reduced the concentration by a factor of 10. Between 10 pM and 10 nM, the target miR-10b concentrations were diluted and the LOD was found to be 100 pM on the surfaces of the gold substrate and the CP-cy3/GS. The substrate’s suitability for producing the LSPR signal remained constant at 3.3 mM, and the gold concentration remained steady even as the levels of miR-10b increased. The LSPR shift steadily increased as the miR-10b concentration rose. This newly developed LSPR probe is crucial for the precise, unmistakable, and simple detection of oncogenes. It is a boon for the biomedical industry, which is now in use and will become more sophisticated in the coming years.

Rostami et al. [73] identified graphene nanoribbons and AgNPs combined with improved LSPR performance as a colorimetric sensor for the simultaneous detection of glutathione and dopamine (DA), utilizing nanocomposites fusion of two hybrid plasmons. It was feasible to effectively detect both DA and glutathione (GSH) at low concentrations, namely 0.04 M for DA and 0.23 M for GSH, using the GNR/AgNPs hybrid as a highly sensitive colorimetric sensor. LODs for DA and GSH were lower when GNRs were utilized (0.1 M for DA and 0.3 M for GSH) compared to when GNRs were absent (0.46 M for DA and 1.2 M for GSH). The experiment demonstrated that the incorporation of GNRs improved the sensitivity of the sensing method compared to when GNRs were not used. Good linearity between a standard calibration curve for DA ranging from 0 to 100 M was employed to generate the hybrid (A525/A625), allowing for the determination of DA concentrations within the range of 0.25–10 M. The equation, with a regression coefficient of 0.999 and an estimated LOD of 0.04 μM, was found. GNR showed an absorption band at 245 nm, while the UV spectrum of the pure multi-walled carbon nanotubes (MWCNT) exhibited absorption peaks at 240 nm and 256 nm. The plasmonic absorption band at wavelengths 330 nm, 400 nm, and 630 nm was found in the LSPR spectrum of AgNP. In the LSPR spectrum of AgNP, there is a noticeable blue shift decreased absorbance intensity from 625 nm to 578 nm, while DA shifted from 625 nm to 525 nm. By detecting the DA and GSH in real serum samples, they illustrated that relative recoveries for DA between 92%-1,075% were attained with RSD% under 5.8%. Additionally, the quantitative GSH recoveries in the serum samples fell between 92%-102.5%, with an RSD% of less than 6.2%. In this work, a quick, simple, and accurate colorimetric sensor having improved performance was developed. The hybrid of GNR/AgNPs was used to find out effective consecutive detection of DA and GSH in serum samples. Additionally, the positive results indicated the potential of the suggested sensor as a probe for sensing clinical diagnostics and biological techniques.

Chen et al. [74] explored the simultaneous identification of breast cancer biomarkers using a plasmonic sensor composed of gold nanorods. They developed a novel method for detecting levels of copper and the cancer antigen 15-3 (CA15-3) in breast cancer serum using a GNR-based plasmonic sensor. This method utilized the optical signal generated by the immune reaction between CA15-3 and GNR@CA15-3 antibody to rapidly detect the tumor marker CA15-3. Additionally, the presence of copper modified the LSPR of GNR, leading to a direct readout of the optical signal. GNR showed an absorption spectrum which indicated transverse peak LSPR occurring at 520 nm and the longitudinal plasmon wavelength (LPW) situated at 773 nm, and finally the red peak showing successful modification. The range of GNR concentrations, as assessed by absorption intensity, spaned from 0.3 to 0.8. LPW was discovered using the CA15-3 increment. An approximate platform demonstrated a clear linear relationship between the degree of red-shift of LPW and CA15-3 concentrations, observed within the range of 0.0249–0.2387 U/mL. Finally, the research provided a combined detection assay for evaluating breast cancer biomarkers based on multifunctional GNR. They demonstrated that the combined detection assay held a significant potential for various applications in the oncology field, particularly in the early diagnosis and observation of malignant tumors like breast cancer. This assay could be valuable in several ways.

Wang et al. [75] explored a sensitive visual detection method for breast cancer biomarkers using NADH-ascorbic acid mediated gold nano bipyramids (AuNBPs) and a multi-color immunosensor. This method can conveniently identify the disease. Many studies showed that breast cancer biomarkers can be identified by measuring the extracellular domain of human epidermal growth factor receptor 2 (HER2 ECD) in serum. As a biomarker for breast cancer, in addition to creating a method for the growth of AuNBPs mediated by ascorbic acid (AA) that is highly responsive and sensitive to the help of NADH, they developed a very accurate multicolor immunosensor for the highly responsive visual identification of HER2 ECD in serum using AuNBPs as the indicator and an antibody as the recognition agent. The suggested multicolor immunosensor featured more color variations that correlated to concentrations of HER2 ECD and improved resolution. Through naked eye observation, it can detect HER2 ECD as less as 0.5 ng/mL, and by using UV-visible spectrophotometry, it can detect HER2 ECD as low as 0.05 ng/mL. ECD for HER2 in human serum using the immunosensor was effectively identified, with a recuperation rate ranging from 94% to 96% and an RSD% of 5% based on a sample size of five. There is a robust correlation between λ_max_ and AA concentration, as indicated by a regression coefficient of 0.991. When the pH is below 9.0, the peak area of AA increases with rising pH, but it decreases when the pH exceeds 9.5. An excessively high pH (> 9.5) can lead to the hydrolysis of AA. Consequently, it is advisable to maintain the pH of ALP enzymolysis within the range of pH 9.0 to pH 9.5. The suggested multicolor immunosensor featured an increased number of color changes that correlated with HER2 ECD concentrations and a high resolution. The proposed immunosensor displayed an intense multicolor change, had a low threshold for detection by the naked eye, demonstrated high specificity, and showed a strong matrix resistance. It is important to note that by utilizing the appropriate antibody as a recognition probe, this immunosensor can be employed as a general method for the visual detection of multiple cancer biomarkers.

Abdi et al. [76] analyzed and designed LSPR-based sensors using coupled nano-rings for cancer detection. They explored an LSPR-based sensor composed of two interconnected AgNPs on a SiO_2_/Si substrate. The sensor sensitivity, full width at half maximum (FWHM), and figure of merit (FOM) were determined as 393.14 nm/RIU, 53.2 nm, and 7.39 RIU^−1^, respectively. The resonance peak originated from the concentrated electric field within the elliptical region between the nano-rings. The designed sensor as a bio-sensor was capable of effectively detecting different types of cancerous cells from healthy cells, since the RI of a cell that is affected by cancer, is higher than that of a healthy cell. The simulation results showed a good selectivity for each type of cancer, with a FOM of approximately 9.5 RIU^−1^. It showed that the concentrated electric field in the elliptical area between the nano-rings caused a resonance peak in the extinction spectrum.

Zhang et al. [77] carried out a study on black phosphorus@AuNPs nanosheet-based LSPR-enhanced singlet oxygen generation and light absorption for tumor photodynamic/thermal therapy. The research involved the use of plasmonic nanoparticles (AuNPs) and BP nanosheets for cancer phototherapy. The goal of the study was to improve singlet oxygen production and hyperthermia effects simultaneously through LSPR to enhance photothermal therapy (PTT) and photodynamic therapy (PDT) for inhibiting tumor development. Preparation and characterization of BP-PEI/AuNPs nanosheet were conductedfor these experiments, and the synthesized BP nanosheet has a lamellar structure with an average diameter and thickness of 343 and 58.6 nm, and 3.9 and 0.7 nm, respectively. Due to the adsorption of positively charged PEI on the surface of the BP nanosheet, the zeta potential of BP-PEI was considerably modified from 27.8 to 21.4 mV. Researchers are exploring various ways to leverage LSPR-based technologies for improved outcomes in oncology. Here are some potential developments and applications of LSPR in cancer research and treatment.

Ney et al. [78] investigated the use of plasmonic NPs in the terahertz band for skin cancer diagnosis with ultrahigh polarimetric image contrast enhancement. By applying InN NPs coated with Parylene-C to the skin, LSPR effects at the terahertz (THz) range were found to enhance subtle water content changes in tissue linked to early stages of skin cancer, according to numerical modeling. Using these Mueller polarimetry techniques, it is now feasible to detect changes in the tissue water content with great sensitivity. Based on the linear degree of polarization, this study provided a LOD for relative changes in the water content of down to 0.0018%. These outcomes are to be expected given that the NPs were inserted using a technique that caused their concentration to generally be low and to exponentially decline in the tissue. The insertion of NPs into the tissue has a minor impact on the sensitivity to a change in water content because the dispersion is virtually nonexistent. Mueller matrix imaging is based on reflection measurements. The repeatability of the reflection coefficient, which is of the order of 0.001, limits the limit of differentiation between skin tissue patches with slightly varied reflection.

Huang et al. [79] investigated the use of noble metal (NM) NPs for the detection and therapy of hematological malignancies. Currently, novel drug delivery systems (DDS) that combine gold, silver, and platinum-based nanomaterials with other targeted biomolecules or direct individual endocytosis have been used to detect and treat hematological malignancies. The development of efficient and potentially theragnostic methods based on NM NPMs could facilitate the clinical tracking, diagnosis, and management of refractory hematological disorders, through a multidisciplinary approach. Using a green nicotinates arborist-mediated synthetic approach, AgNPs with an average size of 22 nm was created. The THP-1 human leukemia cell lines capacity to maintain vitality was affected by the AgNP concentrations (5–50 g/mL), with an IC_50_ of 33.5 g/mL. Tan’s team was the first to report a straightforward method for synthesizing NIR fluorescent gold nanoclusters (AuNCs) that emit light at 660 nm. These AuNCs were effectively stabilized using a multidentate thioether-terminated polymer known as poly (methacrylic acid) modified with PTMP (PTMP-PMAA). These AuNCs were capable of labeling both HeLa cells adhering to surfaces and Jurkat cells suspended in a solution. What made this discovery particularly interesting was the observation that hematopoietic cancer cells, specifically K562 cells, exhibited a notable inclination to internalize a higher quantity of AuNCs compared to normal cord blood mononuclear cells (CBMC). This discovery has significant potential for the development of diagnostic tools for detecting hematologic malignancies. This is because these AuNCs appear to prefer entering relatively mature cells like granulocytes and lymphocytes, which could enhance the diagnostic accuracy for such conditions. AgNPs with fluorescent glycine dimers caps, ranging in size from 9 to 32 nm, have been developed. The ligand system for these blue fluorescent NPs-ligand combinations was subsequently used to bioimage rat basophilic leukemia cells with a high quantum yield of 5.2 ± 0.1%. LSPR holds significant promise for advancing cancer treatment and diagnosis. Researchers are exploring various ways to leverage LSPR-based technologies for improved outcomes in oncology.

Tiwari et al. [80] pioneered the developmnet of a radiometric plasmonic biosensor intended for the detection of Herceptin in HER2-positive breast cancer. Breast cancer, the most crucial cancer for women, should be treated by using optical biosensors like LSPR. The researchers developed an optical sensor based on the direct interaction of trastuzumab (herceptin (HER)), a monoclonal antibody used in treating HER2-positive breast cancer, with plasmonic NPs). These AuNPs were functionalized with citrate ions, which enhanced direct surface contact with HER antibodies. To further increase the sensor's selectivity and sensitivity, the AuNPs were combined with AgNPs at a proportion that has been adjusted. While AgNPs assist in tracking the impact of interferences on the sensing medium, AuNPs used LSPR to detect the HER antibodies. Response surface methodology (RSM), based on the CCD, was used to improve the three key HER sensing parameters of NP ratio, temperature, and pH. RSD was less than 5%. The selectivity of the device was evaluated by observing the LSPR sensor's response to HER in the presence of other biological molecules with comparable features. The selectivity of the device was evaluated. The main advantages of the developed LSPR-based sensor are its quick reaction time (less than 1 min), selectivity, and simplicity.

Soler et al. [81] developed a label-free nanoplasmonic sensor for the early detection of tumor-associated autoantibodies in colorectal cancer. Autoantibodies, which circulate in the blood and are produced by the immune system as soon as a tumor appears, are becoming important indicators for the early detection of cancer. Using a newly developed nanoplasmonic biosensor, they demonstrated the rapid and label-free detection of colorectal cancer autoantibodies in either serum or plasma. Nanoplasmonic technology achieved LOD of about 1 nM (150–160 ng/mL) and provided sensitive and real-time measurement of autoantibodies with great selectivity and repeatability.

Bellassai et al. [82] refined the biomarker-based analysis, which has a considerable promise for a better understanding of cancer on a molecular scale, in a manner that is both accessible and minimally intrusive. In this study, they discussed and assessed the usefulness and usability of platforms based on LSPR and SPR for the identification of several types of cancer biomarkers in liquid biopsy samples. Excellent accuracy, process simplicity, high sensitivity, and specificity provided by LSPR and SPR offered new validation for their application in cancer therapy.

Kaur et al. [83] proposed the use of a very sensitive fiber-optic sensor with spectrum SPR to detect colorectal cancer in the colon or rectal intestine. The sensor performance was examined using the wavelength interrogation method with wavelength of 400 nm–1,000 nm, using transition metal carbides known as MXenes with the chemical formula Ti_3_C_2_TX based layered materials. As normal and malignant colorectal states have distinct RIs, the sensor design can distinguish between them. Compared to an Au-based sensor, the presence of MXenes in Ag-based sensors increases FOM by almost two times. Ag–Ti_3_C_2_-based sensor is the highest previously recorded fiber-optic SPR sensor that has been developed, showcasing an impressive FOM at 76.90 refractive index units (RIU) and an exceptionally high sensitivity of 10,766.28 nm per RIU, primarily designed for the detection of colorectal cancer. It demonstrates the normalized transmitted power spectra for both healthy and malignant colorectal tissues with an Ag-based sensor at three different angles between 400 and 1,000 nm. Three distinct incidence angles of 69, 72, and 78 are used to plot SPR curves. Incident angle has a significant influence on the ability to distinguish between healthy and malignant colorectal tissues. Wavelength shift reduces from 30 nm to 10 nm while moving from 69 to 78. According to the study of angular variation, a lower incidence angle causes SPR to shift to a higher wavelength, which increases sensitivity. Therefore, it is possible to obtain excellent performance from the MXenes-based sensor to precisely detect the RIs of normal and colorectal tissues by regulating the incidence angle and metal thickness. Ag-MXenes and Au-MXenes fiber-optic SPR sensor findings are compared and studied. In the presence or absence of MXenes, Ag-based sensors can offer greater sensitivity and FOM values than Au-based sensors.

Mahani et al. [84] introduced a molecular dynamic and experimental investigation on utilizing hydrogen bonds between prostate-specific antigen (PSA) and antibodies for early-stage prostate cancer detection through LSPR biosensing. PSA aids in the early detection of tumors. Among all PSA detection methods, LSPR is one of the most well-known techniques. This ultrasensitive label-free nanobiosensor was developed for PSA assessment at low blood concentration levels utilizing AgNP. When a probe binds to an antigen, a slight change in the dielectric medium RI causes the LSPR peak shift, which is the sensing mechanism. Here, the anti-PSA antibody is covalently coupled with 10 mm AuNPs, and influencing variables such as the ratio of antibody concentration to AuNPs, pH, and temperature were tuned. The calibration sensitivity was 43.75 nm/(ng/mL), with LOD of 0.2 ng/mL. Damborský et al. [85] studied SPR application in prostate cancer biomarker research, demonstrating that sensor-based techniques can be used to diagnose prostate cancer (PCa) with success. SPR, one of the most quickly evolving methods for determining the kinetics and quantitative binding affinities of interactions between antigens and antibodies in real time, was selected as the best instrument for this purpose. As free or complexed PSA was injected into the dual-flow channels of the SPR device, a variety of PSA-specific antibodies were covalently bound to the biochip surface through amine coupling. Cross-reactivities of the used antibodies, as well as the kinetic parameters and affinity constants of these interactions, were calculated. The SPR biosensor exhibited a linear response to free PSA up to 25 nm. All of these discoveries are necessary for the right design of a lab-on-a-chip that can diagnose PCs using a selective, sensitive, and highly reliable biosensor.

Li et al. [86] proposed a label-free biosensor based on LSPR utilized for the detection of serum interleukin (IL)-10 in individuals diagnosed with endometrial cancer. They fabricated LSPR silver nano structure-based immune sensor and detected cytokine IL-10 in the serum of individuals with endometrial cancer. The fabricated LSPR sensor chip measures the quantification of recombinant IL-10 protein levels, followed by scanning electron microscopy (SEM) and atomic force microscopy (AFM) characterization. Subsequently, rapid detection of endometrial cancer and benign uterine disease serum is achieved using an LSPR sensor within a 30-min. The maximum IL-10 signal in the endometrial cancer group was higher than in the benign group, with shifts of 15.30 ± 6.22 nm versus 8.13 ± 2.57 nm, respectively. The initial size of the unchanged silver nanochip was measured at 619.92 nm. After being exposed to mercaptoundecanoic acid (MUA) overnight, it increased to 635.4 nm, resulting in a 15.48 nm shift towards the red spectrum. Subsequently, following MabIL-10 immobilization, the maximum size reached 658.07 nm, exhibiting a significant 22.67 nm increase. To assess the presence of the cytokine IL-10 in serum samples from 18 individuals (comprising 9 with endometrial malignancies and 9 with benign uterine conditions), both LSPR sensors and enzyme-linked immunosorbent assay (ELISA) kits were employed simultaneously. A *t*-test analysis demonstrated that the LSPR signal (maximum) in the endometrial cancer group was significantly higher than that in the benign group. In summary, the LSPR triangular silver nanostructure biosensor shows promise as a platform for serological detection of endometrial cancer due to its favorable characteristics. However, further refinements are necessary before its application in clinical settings can be considered.

Yuan et al. [87] introduced the serum human epididymis secretory protein 4 from ovarian cancer patients by using a label-free biosensor based on LSPR. The LSPR maximum of the bare silver nanochip was determined to be 592.58 nm before modification. After being modified with 11-mercaptoundecanoic acid, the silver nanochip had a typical LSPR maximum of 619.85 nm and a matching LSPR maximum of +27.27 nm. After anti-HE4 immobilization, the LSPR λ_max_ shifted to 630.97 nm, with an additional 11.12 nm red shift. After incubation with 500 pM HE4, the LSPR wavelength shifted to +14.48 nm, showing a λ_max_ of 645.45 nm. Stepwise increases in LSPR maximum values occurred when HE4 concentrations rose. The curve exhibited a sigmoid shape instead of being linear, as is the case with many immunoassays. The LSPR biosensor offers several benefits for label-free biomarker detection, and gynecological oncology saw the first use of a speciallydesigned LSPR system in diagnostic procedures. The tests in this study show that a label-free LSPR methodology might be a very effective replacement for the traditional ELISA method’s label-based design.

Zhao et al. [88] studied a reusable LSPR biosensor for the quantitative detection of serum squamous cell carcinoma antigen in cervical cancer patients based on an array of AgNPs. They introduced a brand-new, reusable LSPR biosensor for the detection of squamous cell carcinoma antigen (SCCa). First, they used the nanosphere lithography technique, producing a triangular array of AgNPs. Then, by employing AgNPs and monoclonal anti-SCCa antibodies, a functionalized chip surface was made. They evaluated and confirmed the viability of the amino coupling approach for the specific detection of SCCa. The highly sensitive and precise LSPR system was used to effectively evaluate various SCCa concentrations in buffer and human blood, with a linear quantitative detection range of 0.1–1000 pM under optimal circumstances. The LSPR biosensor had good manufacturing repeatability with the use of a suitable regeneration solution, such as 50 mM glycine-HCl of pH 2.0, which reduced both production costs and testing time. The LSPR maximum of the naked silver nanochip was 573.92 nm before modification. On introducing MUA to the silver surface, the LSPR peak experienced a shift of +15.18 nm, reaching 589.1 nm. Subsequent attachment of anti-SCCa monoclonal antibodies caused another red-shift of +13.28 nm, resulting in a detection peak at 602.38 nm. Finally, when 100 pM SCCa was introduced, an additional shift of +9.77 nm was observed, leading to a characteristic LSPR peak at 612.15 nm. LSPR biosensors possess several advantageous qualities, including speed, sensitivity, specificity, label-free operation, reliability, repeatability, affordability, portability, and user-friendliness.

Mariani et al. [89] reviewed the application of SPR and LSPR in clinical analysis, particularly in ovarian cancer. They used the target analyte as an exosome and found a human ovarian cell culture line, and incorporated GNR/AgNP. Narayan et al. [90] demonstrated a protein functionalized SAM-based biosensor for colon cancer detection. They developed a label-free immunosensor based on SPR for continuous monitoring of endothelin-1 (ET-1), a biomarker for colon cancer. A gold disk, modified with a SAM of 11-MUA, was functionalized by covalently attaching monoclonal anti-ET-1 antibodies. The resulting immunosensing platform (ethanolamine/anti-ET-1/11-MUA/Au) underwent characterization using AFM, contact angle (CA), and Fourier-transform infrared (FT-IR) spectroscopy. The SPR electrode successfully detected ET-1 across a broad concentration range of 2–100 pg/mL, achieving a low LOD of 0.30 pg/mL and a high sensitivity of 2.18 pg/mL. Analysis of serum samples from colorectal cancer patients showed results consistent with ELISA findings. Additionally, electrochemical studies validated the effectiveness of the fabricated platform as a point-of-care device for ET-1 detection. Pai et al. [91] proposed a simplified approach for the fabrication of LSPR sensors based on gold nanorods functionalized using mixed polyethylene glycol layers for the activated leukocyte cell adhesion molecule (ALCAM) cancer biomarker. They showed that GNRs bioconjugated with monoclonal antibodies can be easily covalently attached to silanized glass substrates, resulting in highly sensitive LSPR sensors. To enhance the sensor performance, a combination of polyethylene glycol adlayers was optimized for the bioconjugation of monoclonal antibodies using standard carbodiimide chemistry. Under optimal conditions, the ALCAM GNR LSPR sensors exhibited a sensitivity of 330 nm per RI and could detect ALCAM antigen concentrations as low as 15 pM. This uncomplicated fabrication approach has the potential to promote the use of LSPR sensors in the field of immunoassays [91]. These attributes make LSPR methodology particularly valuable compared to alternative immunoassay methods for the detection of various cancer biomarkers (Table 1) [[68], [69], [70], [71], [72], [73], [74], [75], [76], [77], [78], [79], [80], [81], [82], [83], [84], [85], [86], [87], [88], [89], [90], [91]].

**Table 1:** Applications of LSPR in the detection and management of cancer.

S. No.	Target analyte	Sample matrix	Technology (detection method)	Cancer type	LSPR shift	Detection limit	Sensitivity	Sample	Regression coefficient	Refs.
1	Methotrexate	FA-AuNPs	Assay based LSPR	Not specific	–	155 nM	High	Blood/tissue	–	[68]
2	Nanoparticles with HeLa cells	GNR–PANI	GNRs coating with biocompatible polyaniline (PANI)	Photothermal cancer	808 nm	650–900 nM	Moderate	Tissue	0.999	[69]
3	Human alpha-fetoprotein and prostate-specific antigen	An optofluidic device with SAM, PDMS substrate alignment	Lab-on-a-chip technology of LSPR	Cancer markers (not specific)	–	5−1,000 ng/mL	High	Serum	0.998	[70]
4	Prostate-specific antigen and human alpha-fetoprotein	Nanoparticles composition with Fe^3+^ at room temperature	The vacancy of Cu_2−x_Se nanoparticles was tuned by doping with magnetic ferric ions	Photothermal therapy for cancer	–	–	High	Serum	–	[71]
5	MIR-10b	GNR/AgNP	LSPR	Stomach cancer	–	–	High	Blood/tissue	–	[72]
6	Ggraphene Nano ribbons	GNR/AgNP	LSPR	Neuroblastoma	625 nm	0.04 μM	–	–	–	[73]
7	Copper and the cancer antigen 15-3 (CA15-3)	GNR/AgNP	Assay based LSPR	Breast cancer	520 nm	–	–	Serum	0.998	[74]
8	Gold Nano bipyramids	GNR	LSPR	Breast cancer	773 nm	–	–	Serum	–	[75]
9	Different types of cancerous cells from healthy cells	GNR/AgNP	LSPR-based sensor consisting of two coupled silver nano-rings on a SiO_2_/Si substrate	Not specific	393.14 nm	–	–	Blood/tissue	0.998	[76]
10	Different types of cancerous cells	Black phosphorus@AuNPs nanosheet-based	LSPR	Tumor photodynamic/thermal therapy	343 nm	–	–	Tissue	–	[77]
11	Changes in the tissue’s water content	Plasmonic nanoparticles (NPs)	Nanoparticles coated with Parylene-C	Skin cancer	–	–	High	Tissue	0.998	[78]
12	THP-1 human leukemia cell lines	Poly (methacrylic acid) modified with PTMP (PTMP-PMAA) nanoparticles	Green nicotinates arborists-mediated synthetic approach	Hematological malignancies.	660 nm	–	High	Serum	–	[79]
13	Herceptin in HER2-positive breast cancer	Citrate ions were used to adorn AuNPs	LSPR	Breast cancer	–	600–800 nM	High	Tissue	0.998	[80]
14	Different types of colorectal cancerous cells	GNR/AgNP	LSPR	Colorectal cancer	–	150–160 ng/mL	High	Serum/plasma	–	[81]
15	Several types of cancer biomarkers	GNR/AgNP	LSPR	Not specific	–	–	High	Blood/tissue	0.998	[82]
16	RI difference between normal & colonial tissue	Mxenes, Ag	Fiber optic LSPR biosensor	Colorectal cancer	1,000 nm	FOM 76.90	10766.28 nm/RIU	Colorectal tissue	–	[83]
17	Antibody, PSA	Gold nanoparticles 10 mm	LSPR	Prostate cancer	–	–	–	–	0.998	[84]
18	PSA-specific antibodies	Lab-on-a-chip LSPR	LSPR	prostate cancer	–	–	High	Tissue	0.9974	[85]
19	Serum interleukin-10	Silver nanoparticles HUNPs	LSPR	Endometrial cancer	635.4 nm	–	–	Serum	–	[86]
20	Serum human epididymis secretory protein-4	Silver nanochip +11-mercapto undecanoic acid, anti HE 4 immobilizations	LSPR	Ovarian cancer	619.85 nm, 630.97 nm	10–10,000 pM	–	Serum	0.998	[87]
21	Squamous cell carcinoma antigen (SCCa)	AgNP, monoclonal anti-SCCa antibody	LSPR	Cervical cancer	589.1 nm, 612.15 nm, 602.38 nm	0.1–1,000 pM	High	Cervical tumor cell line	–	[88]
22	Exosome	GNR/AgNP	LSPR	Ovarian cancer	–	–	–	Human ovarian cell line culture	–	[89]
23	GTF2b &ED 0.3	Gold/Zno/MHDA/TAA	LSPR	Colorectal cancer	70 nm	50 ng/mL, 10 ng/mL	Low	Colorectal tissue	–	[90]
24	ALCAM	Mixed/PEG/GNR	Label based LSPR	Colorectal cancer	–	0.25 nm–25 nm	High	Serum	0.998	[91]

–: no data; FA-AuNPs: gold nanoparticle modified with folic acid; LSPR: localized surface plasmon resonance; GNR: gold nanorod; PANI: polyaniline; SAM: self-assembled monolayer; PDMS: polydimethylsiloxanes; MIR-10b: micro RNA 10b; GNR/AgNP: silver nanoparticles and graphene nanoribbons;RI: refractive index; PTMP-PMAA: pentaerythritol tetrakis 3-mercaptopropionate-polymethacrylic acid; PSA: prostate specific antigen; RIU: radio interface unit; ALCAM: activated leukocyte cell adhesion molecule.

Wang et al. [92] introduced a fiber-optic sensor probe for detecting cardiac troponin I (cTnI) using a 2D nanomaterials-enhanced LSPR fiber biosensor designed for the identification of cardiac troponin 1(cTn1). To enhance both the stability and sensitivity of the sensor, a combination of graphene oxide (GO), AuNPs, and molybdenum disulfide NPs (MoS_2_-NPs) was affixed onto the etched metal-coated plastic (MPM) surface. An enzyme was introduced to the sensing interface to improve the overall performance of the sensor. The absorption spectrum of the nanomaterials was evaluated using UV spectroscopy and transmission electron microscopy (TEM), while SEM was used to visualize the surface-bound probe. The experiment’s sensitivity was determined as 3.4 pm/(ng/mL), with a correlation coefficient of 0.928. The LOD was established as 96.2638 ng/mL. Regarding the sensor’s ultimate performance characteristics, the identified linear range spanned from 0 to 1,000 ng/mL. LSPR plays a crucial role in measuring cardiac troponin levels and is widely utilized with strong efficacy in the biomedical domain. Furthermore, this detection principle holds universal applicability for real-time analysis of diverse targets in fields like environmental conservation, food safety, and homeland security. Tang et al. [93] proposed the measurement of cardiac biomarkers for diagnosing myocardial infarction through the utilization of label-free and multiplexed gold nanorod bioprobes. The aspect ratio and peak wavelength of LSPR were shown to be directly correlated. They developed a multiplexed sensor using GNRs with aspect ratios of 2.1 and 4.2 by integrating certain antibodies. These GNRs displayed longitudinal plasmonic bands with individual LSPR wavelengths at 640 nm and 830 nm. These nanorods were combined, and changes in analyte concentrations were deduced by detecting spectrum shifts. This was done by measuring the spectrum shift at various plasmon band peaks after the antibodies specifically bound to those points. The practical application of these combined bioprobes was outlined for the simultaneous measurement of biomarkers of heart, including cTnI and myoglobin, in a range relevant to clinical diagnosis. It was discovered that the degree of the red shift in the LSPR had a direct linear connection with the rise in the concentration of the desired analytes. This relationship was quantified through a calibration curve, resulting in a regression coefficient of 0.98. Manipulating the aspect ratio of the nanorods led to a lengthened LSPR absorption, manifesting as a plasmonic peak spanning from 600 to 1,100 nm. The stronger longitudinal band pertains to electron oscillation along the nanorod’s longer axis, while the transverse band at around 520 nm signifies gold’s characteristic absorption. The longitudinal band’s absorption can be flexibly adjusted across the visible to NIR spectrum. UV-visible spectroscopy was employed to track the displacement of plasmonic band peaks towards longer wavelengths. Given the heightened sensitivity of longitudinal SPR to alterations in the local RI, the shift in longitudinal spectra garnered emphasis in this label-free plasmonic biosensing approach. It is anticipated that this downscaled nanosensor will significantly streamline the on-site assessment of numerous biomarkers from a single sample, enhancing the speed and dependability of medical diagnostics.

### 6.2 Microbial biosensing of LSPR

Maphanga et al. [94] developed a diagnostic methodology. A novel optical biosensor chip was created and put to the test utilizing LSPR to track the interactions between the anti-mycobacterium tuberculosis antibody and the mycolic acid TB antigen. Mycolic acid was successfully attached to a biosensor chip that was coated with gold. A goat anti-rabbit IgG H&L secondary antibody was successfully utilized in conjunction with AuNPs to improve the detection signal brought on by biomolecular binding interactions. Characterization was performed using UV spectroscopy where the antibody was successfully linked to the AuNPs, leading to a reduction in absorption intensity in the TB-bioconjugate. This change indicated the successful binding of the antibody to the AuNPs. This binding is crucial as it enhances the RI on the biosensor chip’s surface, thereby improving the performance of the biosensor. The absorption spectra of the AuNPs showed that they had an absorption peak at 575 nm before binding to the antibody, which shifted to 585 nm after the binding, confirming the successful conjugation. This shift of approximately 10 nm provided further evidence of the effective bioconjugation process. It was done using Dynamic Light scattering (DLS) to examine the distribution of particle sizes. Based on intensity, this method detected changes in the size distribution. The main objective was to study the AuNPs both before and after their conjugation with the secondary antibody. At a temperature of 25 °C, each sample was exposed to three different measurements (in triplicates), which were then repeated three times (*n* = 3). The dispersant had a RI of 1.330 and a viscosity of 0.8872 cP. Three types of sample groups were examined: unmodified AuNPs, TB-bioconjugates (produced as a result of the bioconjugation procedure), and TB-bioconjugates in the presence of mycolic acid. Characterization was also performed by FT-IR spectroscopy, SEM & energy-dispersive X-ray (EDX) spectroscopy, and AFM. A customized biosensing system utilizing LSPR was effective in distinguishing between experimental and control groups in the context of detecting anti-mycobacterium tuberculosis antibodies. During LSPR biosensing, in terms of peak intensity and the area under the peaks, the reference sample exhibited greater light transmission, followed by the control samples, whereas the experimental sample demonstrated reduced light transmission. This collectively demonstrated the potential of mycolic acid as a valuable biomarker for capturing antibodies against mycobacterium tuberculosis. This discovery holds promise for the creation of biosensor chips tailored for tuberculosis diagnosis. Furthermore, optical detection methods like LSPR can reliably analyze these biosensor chips for diagnostic applications.

Wei et al. [95] derived plasmonic photocatalysts for biosensing of microbes using LSPR technology. The literature on the utilization of plasmonic photocatalysis in microbiological contexts was examined. This encompassed its applications in antibacterial, antiviral, and antifungal domains, along with a novel investigation into its potential for other microbiological functions. Some tests may have been carried out with UV irradiation present, encompassing indoor light, natural sunlight, or artificial sources. Consequently, diverse mechanisms of microorganism inactivation should be considered. These mechanisms include the direct activation of wide-band gap semiconductors through UV exposure and the excitation of plasmonic effects under visible light. Concerning plasmonic excitation, the transfer of “hot” electrons is anticipated, characterized by electron movement from noble metals to semiconductors under visible light, and the reverse process under UV, where electrons transfer from semiconductors to noble metals. Alterations in the oxidation state of less noble metals like silver (Ag) and copper (Cu) are likely. This could facilitate the adsorption of photocatalysts onto microorganism surfaces while possibly resulting in the release of metal cations into the solution or suspension. For comprehensive mechanism analysis, it is advisable to carry out tests under exclusive visible light conditions (similarly under exclusive UV conditions for comparison) and in complete darkness. This can be guided by action spectrum analysis. Plasmonic photocatalysts also hold considerable potential for various microbiological applications, particularly in cancer treatment and anticancer therapy. However, a greater emphasis on research is needed to enhance the specificity of targeting only malignant cells while sparing healthy cells [96].

The LSPR nano biosensor’s great sensitivity makes it a potential elegant substitute for detecting pathogen microorganisms that are present in trace amounts in food and beverage samples. Manzano et al. [97] developed biosensors using LSPR technology to identify the presence of Brettanomyces bruxellensis in wine. In this work, a thiol-modified DNA probe was anchored at its 5′ end to a nanostructured gold surface. To develop a nano biosensor based on the troublesome wine yeast Brettanomyces bruxellensis, LSPR technology was used. The gold layer was evaporated to a thickness of 4 nm. 2 L samples of DNA from the target microorganism and negative control were used at various concentrations to gauge the technique’s sensitivity and specificity. The interaction between the NPs and the DNA probe results in changes in the optical properties of the particles, which shift the peak of LSPR extinction (λ_max_). The results, which used *Saccharomyces cerevisiae* as the negative control and B. bruxellensis as the target microorganism, highlighted the accuracy of the DNA probe and the procedural method. DNA targets may be effectively identified using LSPR spectrophotometry at concentrations as low as 0.01 ng/L. This demonstrates the potential use of this technique for identifying minute quantities of harmful bacteria in samples of food and beverages. On TEM grids, four patterns were used to evaluate the usual extinction spectrum of NP samples without alterations. Three separate grids were used for this examination, which resulted in an optical density (OD) value of 0.2094 and an average wavelength of 552.76 nm. A distinct red shift in resonance frequencies was seen after each occurrence of biofunctionalization using the DNA-probe complex on AuNPs compared to the unmodified NPs. The resultant mean wavelengths for the DNA samples in the presence of Tris-HCl were 557.43 nm, 566.33 nm, and 574.45 nm, respectively, for PBS and saline-sodium phosphate-EDTA buffer (SSPE). These findings suggest a variation in the displacement of the peak, with shifts of 4.67 nm, 13.57 nm, and 21.69 nm observed when employing AuNPs for Tris-HCl, PBS, and SSPE buffer, respectively. The LSPR spectrophotometry method verified that 0.01 ng/L DNA targets could be detected. Using the genomic DNA recovered from the pure cultures and without the need for probe labeling techniques, a clear distinction was established between the specific hybridization with DNA forms.

Taghipour et al. [98] developed MIM nano-discs utilizing highly sensitive LSPR for rapid identification of severe acute respiratory syndrome coronavirus 2 (SARS-CoV-2). MIM nanostructures made of Au/SiO_2_/Au layers, serving as biosensors, were the subject of a three-dimensional FDTD simulation. The Au/SiO_2_/Au cavity’s strong interaction between the twin plasmonic contacts allowed for much higher sensitivity. The binding resulted in an alteration of the RI, enabling the detection of coronavirus disease 2019 (COVID-19). Due to the increased overlap between the plasmonic mode and the nanodiscs surroundings, heightened sensitivity of 312.8 nm/RIU was achieved. Pinnacle wavelength of the suggested configuration experienced a shift of approximately 47 nm when the RI of the surrounding medium transitioned from 1.35 (absence of binding) to 1.5 (complete binding). The alteration in the intensity of the SPR peak can serve as an additional sensing mechanism for the detection of SARS-CoV-2. Ultimately, the performance of the biosensor was assessed by employing the refractive index modifications previously documented for different concentrations of the SARS-CoV-2 S-glycoprotein solution. To assess the sensor’s sensitivity, three scenarios of SARS-CoV-2 binding were taken into account. Through the manipulation of the MIM layer dimensions, an absorption peak wavelength of 715 nm was achieved when no SARS-CoV-2 viruses were bound. Subsequently, the peak wavelength transitioned from 715 nm in the absence of binding to 739.5 nm and 759.5 nm for moderate and complete SARS-CoV-2 binding, respectively. Elevated concentrations of COVID-19 corresponded to more significant shifts in the RI within the surrounding medium. The relationship between resonance wavelength shifts and the RI of the encompassing medium for the Au nano-disc demonstrates a linear equation with a regression coefficient of 0.996. The sensor’s design offers an efficient means for swiftly detecting SARS-CoV-2. The plasmonic waves exhibit an exponential decay across the two Au/SiO_2_ interfaces, leading to an elevated resonance peak and consequently heightened sensitivity (measured at 312.8 nm/RIU). A shift of 47 nm in the peak wavelength occurred as the refractive index of the surrounding medium transitioned from no binding at 1.35 to complete binding at 1.5. Employing previously established alterations in RI for various based on fluctuations in concentration, the concentrations of the SARS-CoV-2 S-glycoprotein solution, the sensitivity, and the FOM were calculated.

Oh et al. [99] proposed AuNP aptamer-based LSPR sensing chips for the rapid detection of *Salmonella typhimurium* in pork meat. The aptamers that were integrated into the LSPR sensing chips demonstrated an extremely sensitive upper LOD of around 10⁴ CFU/mL for *S.* t*yphimurium* when tested in a pure culture setting under the optimal assay conditions. The entire analysis process took approximately 30–35 min. Moreover, the LSPR sensing chips were also utilized on pork meat samples artificially contaminated with *S.* t*yphimurium*, yielding an LOD of 1.0 × 10^4^ CFU/mL. Notably, the devised approach could identify *S. typhimurium* in spiked pork meat samples without the need for a pre-enrichment step. Furthermore, the LSPR sensing chips created for detecting *S.* t*yphimurium* remained unaffected by the presence of a food matrix or any background contaminants in the microflora. This newly developed LSPR sensing chip holds promise for identifying harmful pathogens in settings such as chemical and clinical laboratories, the agricultural sector, the food industry, and environmental monitoring.

### 6.3 Miscellaneous category

Tambe et al. [100] described fluoride impurity sensing in potable water using an LSPR-based fiber optic sensor. They showed the photochemical reaction induced by light beam from He–Ne laser wavelength 633 nm, which was focused on bare core immersed in the solution of silver nitrate and sodium citrate. They observed that following laser exposure, there was growth and accumulation of AgNPs on the fiber core. The process of laser activation led to the reduction of silver particles within the illuminated solution. This illumination process lasted for 6 min. The resultant deposited NPs displayed irregular shapes and had diameters reaching up to 50 nm. When distilled water was present, a trough with a wavelength of 541 nm was visible in the output spectrum. This trough’s location changed after a fluoride solution with a known concentration was added. For three different known amounts of fluoride dissolved in distilled water – 30 ppm, 50 ppm, and 100 ppm – the intensity of the output was graphed. It was noted that the SPR dip showed a shift towards longer wavelengths when the fluoride impurity concentration rose. A fiber optic sensor was developed utilizing LSPR to identify fluoride impurities within water. The deposition of AgNPs was achieved through a straightforward and cost-effective method involving laser-induced NP deposition. The practical outcomes of the experimentation underscore the efficacy of this approach for the detection of water impurities.

Fattahi et al. [101] performed amplification of LSPR through the utilization of magnetic nanoparticles (MNPs) to achieve highly sensitive bioanalytical assays in human blood plasma. They demonstrated how Fe_3_O_4_ MNPs can enhance metal NPs’ LSPR. This improvement was attributable to Fe_3_O_4_ MNPs’ considerable refractive index and molecular weight, which supported better binding interactions in biological processes. The study included a label-free LSPR nanosensor for locating disease biomarkers in physiological fluids, providing a practical and affordable detection technique. To assess the practical efficacy of Fe_3_O_4_ MNPs in enhancing LSPR assays, they employed cardiac troponin I as a model protein for diagnosing myocardial infarction. A GNR bioprobe was used to detect cTnI, and when cTnI molecules captured by MNPs were compared to direct cTnI adsorption on the GNR sensor, the spectral responses were up to six times stronger. The LOD for plasma samples was notably lowered to around 30 pM, surpassing similar studies by three orders of magnitude. The absorption spectrum of the GNRs typically exhibits two distinct SPR peaks, as shown in the inset of panel A. The initial SPR peak occurs at 520 nm and corresponds to the transverse electron oscillation of gold. The second SPR peak appears at 650 nm, which is attributed to the longitudinal electron oscillation along the length of the rods. This second peak becomes more pronounced at longer wavelengths and varies based on the aspect ratio of the rods. By adjusting reaction parameters, such as the concentration of silver ions and the seed-to-Au^3+^ ratio, it was possible to synthesize GNRs with adjustable LSPR wavelengths ranging from 650 to 1,050 nm, corresponding to aspect ratios of 1.5 to 8.9, respectively. To perform on-site analysis of trace target molecules for medical diagnostics, environmental protection, food safety, and homeland security, this ultrasensitive nanosensor is highly appropriate to be combined with a small optical detector.

## 7 Applications of SERS biosensors

### 7.1 Bottom-up created plasmonics biosensing devices for machine-learning biomedical studies

Abalde-Cela et al. [102] found that SERS possessed the potential to serve as a highly effective sensing platform, enabling its integration into *in situ* biosensors. These biosensors can continuously monitor the microenvironment of cells and their communication processes. For SERS, a biocompatible hybrid material was developed by adding gold-based nanostructures to sponge-shaped gellan gum hydrogels. This novel substance was used as a SERS substrate to identify disease-related biochemical indicators in cells. By using these 3D plasmonic polymeric matrices, we were able to prove the detection of two cancer-cell-related extracellular metabolites, lactate and thiocyanate. In this study, achieving a uniform dispersion and distribution of NPs within the GG-SLH was essential. To achieve this, the NPs were incorporated in situ before the precursor hydrogel underwent the gelation reaction. It was difficult to detect these two tiny compounds using SERS, but it became possible because of the extra boost that both kinds of anisotropic NPs offered. Furthermore, by stabilizing the NPs with the gellan gum scaffold, these cutting-edge SERS platforms may be used for real-time growth and metabolic monitoring of 3D cell models.

Ko et al. [103] focused on the integration of a fiber-based cell culture and biosensing system for monitoring multiple protein markers secreted from stem cells. By fusing a SERS-based biosensor with a three-dimensional cell culture scaffold, a new platform was developed. These elements were layered to create a multilayer system that made it possible to monitor the protein markers that were generated by grown stem cells without the need for regular harvesting of cells or media. Protein markers were identified by the SERS capture substrate in conjunction with an Au–Ag alloy nanobox-based SERS tag. The SERS tag’s integration of the many Raman reporters made it simple to identify target proteins for multiplex experiments. They asserted that the developed SERS-based immunoassay could detect protein markers at levels as low as pg/mL without any interference. By combining a culture scaffold for adipose-derived stem cells (ADSCs) with multiple SERS capture substrates and incubating them in differentiation culture media, the system demonstrated high sensitivity. It effectively monitored the time-dependent secretion of three distinct osteogenic protein markers from ADSCs during their process of osteogenic differentiation. The scaffold for cell culture effectively facilitated the growth and differentiation of ADSCs in osteogenic media (OM). Simultaneously, a SERS capture substrate, in conjunction with SERS tags made from Au–Ag alloy NPs, was able to detect various protein markers. By integrating one ADSC culture scaffold with multiple SERS capture substrates designed for specific target markers and incubating them in OM, the system successfully tracked the time-dependent secretion of three distinct markers related to osteogenic differentiation (ALP, OC, and FN) over three weeks. It is feasible to alter the sensor and cell culture scaffold separately, and by combining different cell and biomarker combinations, pertinent data on the true condition of the various cell types may be obtained.

### 7.2 Fabricated plasmonic biosensing devices with uniform hotspot arrays for machine-learning bioanalysis of biosamples

Nam et al. [104] showed refractive-index-insensitive nanolaminate SERS substrates for label-free Raman profiling and classification of living cancer cells. They described that in traditional SERS substrates, the EFs are significantly influenced by the background RI. This dependence poses challenges for conducting accurate spatiotemporal SERS analysis on living cells, especially when these cells have varying extra- and intracellular organelles with diverse local RI values ranging from 1.30 to 1.60. Show that Nano laminated SERS substrates can maintain consistent arrays of vertically aligned nanogap hotspots with high SERS EFs (>10^7^), regardless of variations in background RI. It was discovered via experimental and computational research that the wide and multi-resonant optical properties of Nano laminated plasmonic nanostructures were the source of the SERS response’s insensitivity to changes in the non-refractive index. These Nano laminated SERS substrates, which are insensitive to the RI, were used as a proof of concept to perform label-free Raman profiling and categorization of living cancer cells, achieving a high prediction accuracy of 96%. They discussed schematic descriptions of label-free SERS measurements of living breast cancer and normal cells, the bright-field image, the top-view SEM image, and the cross-sectional SEM image of cultured breast cancer cells on Nano laminated SERS substrates. Their conclusion suggests that refractive index-insensitive Nano laminated SERS substrates with high performance have the potential to facilitate label-free spatiotemporal biochemical analysis of living biological systems.

Lussier et al. [105] suggested nanolaminate plasmonic substrates for high-throughput living cell SERS measurements and artificial neural network (ANN) classification of cellular drug responses. They utilized nanolaminate SERS substrates interfaced with cells to conduct dependable high-throughput SERS measurements. These experiments involved well-studied living cancer cells subjected to four different drug dosages. By employing an ANN for multiclass classification of cellular responses to the drugs, they achieved a high accuracy rate of 94%. Notably, these nanolaminate SERS substrates, possessing a high SERS EF (>10^7^), rapidly generated large SERS datasets, providing extensive molecular information on living cells (10,000 spectra within 3 min). This rich data could be harnessed for machine learning methods that require substantial data inputs. Five other popular machine learning techniques are shown to be less successful in multiclass classification than the ANN. These techniques include support vector machines (SVM), partial least-squares discriminant analysis (PLSDA), classification trees (CT), k-nearest neighbor (KNN), and principle component analysis coupled with linear discriminant analysis (PCA-LDA). ANN performs exceptionally well at identifying extra complex patterns seen in high-dimensional spectroscopic data. By showcasing the application of drugs on living cells, the authors expect that nanolaminate SERS substrates can monitor cellular responses to various external stimuli without the need for labels, offering a non-invasive approach.

Garg et al. [106] researched in situ spatiotemporal SERS measurements and multivariate analysis of virally infected bacterial biofilms using nanolaminated plasmonic crystals. They noted that very few studies have been conducted on multivariable analysis of spatiotemporal SERS datasets to extract spatially and temporally correlated biological information from multicellular systems. The researchers conducted real-time label-free SERS measurements and sophisticated multivariate analysis of *Pseudomonas syringae* *(P. syringae)* biofilms. This analysis was done during their growth stages and when they were infected by the bacteriophage virus Phi6. They achieved this by utilizing nanolaminate plasmonic crystal SERS devices, which interfaced with mechanically stable, consistent, and densely distributed hotspot arrays within the *P. syringae* biofilms. They employed PCA and hierarchical cluster analysis (HCA), two unsupervised multivariate machine learning approaches, to examine the Phi6 dose-dependent fluctuations and spatiotemporal changes in prominent Raman peaks inside *syringae* biofilms. These peaks were linked to biological constituents such as metabolites, extracellular polymeric substances (EPS), cellular components, and extracellular medium loaded with cell lysate. They applied supervised multivariate analysis through LDA to classify biofilm responses based on different Phi6 doses. This showcased the capability of diagnosing viral infections by expanding the real-time SERS method to observe diverse interactions between viruses and bacterial networks. This approach holds potential in various applications, including the development of phage-based therapies against biofilms and the continuous detection of pathogenic viruses.

Tang et al. [107] introduced the utilization of CRISPR-Cas12a within a SERS lateral flow assay (LFA). This innovative approach enabled the direct recognition of both double-stranded DNA and single-base mutations, eliminating the necessity for DNA amplification. To improve the precision and accuracy of nucleic acid detection in LFAs, a new strategy combining CRISPR-Cas12a with SERS was developed. Direct measurement of HIV-1 double-stranded DNA was accomplished with a LOD of 0.3 fM by combining the very precised SERS markers with the focused signal amplification capacity of CRISPR-Cas12a, doing away with the requirement for any previous amplification steps. Compared to traditional colorimetric LFA methods, this sensitivity improvement is approximately 4 orders of magnitude greater. Additionally, the whole detection procedure may be finished in less than an hour. Utilizing the inherent specificity of Cas12a, even minute quantities of the HIV-1 single-based drug resistance mutation (M184V) at levels as low as 0.01% can be accurately identified. This method is effective not only in controlled buffer conditions but also in serum samples, exhibiting similar performance. As a result, the cost-effective and straightforward paper-based CRISPR-SERS strip holds significant promise for point-of-care nucleic acid target testing, particularly in settings with limited resources or lacking laboratory facilities. Utilizing the HIV-1 double-stranded DNA as the target template, results were achieved within an hour. By leveraging the specificity of Cas12a, even minute amounts of the HIV-1 single-based drug resistance mutation (M184V) were reliably detected at levels as low as 0.01%. The detection accuracy spanned from 98% to 105%, demonstrating a narrow relative standard deviation between 3.5% and 5.2%. The calibration plots for HIV-1 double-stranded DNA displayed a regression coefficient of 0.9857. This technique holds promise as a viable option for point-of-care diagnostics in clinical settings. An additional benefit of this biosensor is its capability for multiplexing and seamless integration within the LFA assay. This enables the simultaneous analysis of multiple samples by incorporating numerous strips onto a single pad.

Pang et al. [108] researched the application of SERS for environmental detection. Proper utilization of SERS for identifying organic pollutants stands out as an emerging application that contributes to both human health and environmental protection. Addressing pharmaceutical contaminants within water systems requires the implementation of biosensing techniques. Pharmaceuticals, such as painkillers like tramadol and desvenlafaxine, can lead to unintended biological alterations in non-target organisms. Employing SERS as an analytical detection tool, substances like titanium dioxide and tungsten disulfide, along with photocatalytic degradation agents, demonstrate the potential to achieve removal rates ranging from 68% to 100% for NSAIDs, antibiotics, and cosmetic dyes. Remarkably, for rhodamine B, the LOD was as low as 10 nm, accompanied by a removal efficiency of 100%. SERS’ exceptional sensitivity and non-destructive nature make it an excellent choice for plastic detection. It has been proven to be capable of identifying particles within complex sample combinations and enables the identification of particles as tiny as nanometers. SERS has a lot of potential as a technique for precisely identifying synthetic dyes at minuscule concentrations. Standard tannin foam, glyoxal tannin foam, and TWEEN Pluronic tannin foam were created as three unique foam substrates. A 40-nm-thick silver coating was forced to apply a magnetron sputtering technique to coat or deposit materials onto these substrates. The resulting signals were less intense on the common tannin foam. Compared to the signals from ordinary tannin foam, the intensity on TWEEN Pluronic tannin foam was noticeably raised by a factor of 23.36, and the relative standard deviation was reduced by 36%. Yang and colleagues presented a method for synthesizing porous octahedral Cu_2_O onto Cu MOFs, resulting in the innovation of a distinctive SERS substrate. The sensitivity of SERS was remarkably elevated using this recently devised substrate, enabling the successful detection of methylene blue (MB) at extremely minimal concentrations of 5 × 10^−9^ M.

Nam et al. [109] proposed plasmonically calibrated label-free SERS for improved multivariate analysis of living cells in cancer subtyping and drug testing. SERS has become a fast and non-invasive method for characterizing molecular fingerprints in intricate biological samples. Nevertheless, label-free SERS analysis encounters difficulties in consistency and accuracy. This is because SERS signals are highly influenced by fluctuations in local optical fields at plasmonic hotspots. These variations can distort the connections between the observed spectroscopic characteristics and the true molecular concentration patterns in complex biological matrices. They reported that metal nanostructures’ plasmonically enhanced ERS signals may be used as an internal SERS calibration standard to facilitate multivariate analysis of biological systems in living organisms. They showed that employing ERS-based SERS calibration can improve the classification accuracy of label-free living cell SERS spectra. This enhancement was observed in distinguishing between subtypes of breast cancer cells with varying levels of malignancy and evaluating the responses of cancer cells to different drug dosages.

Kagan et al. [110] directed their efforts toward distinguishing acid red 26 from acid red 18 by employing silver nanostars while examining different conditions of PH. The SERS biosensor presents a convenient means for ensuring environmental safety through the detection of pesticides, herbicides, and combinations of pesticides, as well as biological contaminants such as bacteria, viruses, and mycotoxins. The homogeneity problems with the SERS nano-substrate are a common cause of the inconsistent repeatability in SERS findings. Integrating IS offers a viable path forward for research on SERS is advantageous as it removes the requirement for perfectly uniform substrates and enables precise quantitative analysis of analytes. Considering that real-world samples frequently encompass interfering constituents, the development of SERS techniques is capable of identifying specific substances within complex matrices like soil samples or biological tissues. Although some publications claim that their methods can be applied in real-world environmental situations, it has been discovered that samples in complex matrices with interferences often show reduced Raman enhancement compared to more straightforward laboratory samples [111].

Yang et al. [112] successfully isolated and identified pesticide (carbendazim) residues in genuine food samples, specifically orange juice and kale leaves. They employed a hybrid approach involving on-chip thin layer chromatography (TLC) coupled with SERS spectroscopy to effectively separate and identify carbendazim (MBC) within the intricate food matrices. The substrate of choice for their SERS technique was composed of GANPs, while the target analyte was carbendazim, with a LOD of less than 2 mg/L.

Albarghouthi et al. [113] demonstrated thiram molecules denoted as “K,” on the plasmonic nano-urchins. This quality plays a crucial role in obtaining a SERS spectrum and should be duly considered. They found the LOD of 10 pM. The analyte assembled gold/zinc oxide nano-urchins for SERS sensing of the pesticide thiram. A significant enhancement in the SERS effect has been observed when using assembled Au/ZnO nano-urchins. This notable upgrade can be attributed to the exceptional adsorption amplitude and sample matrix they used during the experiment, which are thiram and analyte on SERS surface respectively.

Shen et al. [114] helped in preparation of Cu_2_O@Ag microspheres resembling Ferrero® chocolate has been done in-situ to act as a substrate for SERS and enable the detection of thiram. This work used a simple in-situ redox reaction to produce Cu_2_O@Ag microspheres that resembled Ferrero® chocolate (referred to as FRC-Cu_2_O@Ag). Cu_2_O served a dual function in the production and growth of AgNPs on the surfaces of these microspheres, acting as both templates and reducing agents. Cu_2_O@Ag was used as the substrate for SERS investigation, especially for the identification of compounds like 4-mercaptobenzoic acid (4-MBA) and thiram. The resultant hybrids comprised semiconductors and noble metals. The LOD was found to be 0.018 mg/L, with apple peels serving as the sample matrix.

Barbillon et al. [115] developed nylon membranes that have been altered with AuNPs and utilized as substrates for SERS to detect various pesticides. They provide a simple SERS method that uses several enhanced techniques to improve the consistency of SERS analysis utilizing regular nylon membranes as substrates for the detection of trace chemicals. To confirm the appropriateness of the substrate, we put this strategy to the test with three different pesticide solutions. Notably, we were able to detect thiram with an outstanding LOD of 108 g/mL, whereas phorate and benthiocarb both had LODs of 106 g/mL. In conclusion, the addition of AuNPs to nylon membrane substrates improves the cost-effectiveness and dependability of SERS, making it more appropriate for extensive commercial application in trace chemical detection.

Yao et al. identified dithiocarbamate, chloronicotinyl, and organophosphate pesticides by employing electrochemical activation to enhance the SERS properties of screen-printed electrodes (SPEs). The electrochemical method of activating metallic SPEs results in the consistent creation of nanostructures that exhibit outstanding SERS characteristics. By electrochemically activating gold SPEs using cyclic voltammetry, it was possible to detect pesticide concentrations as low as µg/L. The development of novel nanostructures with the SERS effect is credited with this detecting capacity. The analyte used was chlorpyrifos and the sample matrix for the experiment was tap water.

Yu et al. [117] reported that a three-dimensional plasmonic crossed-wire nanostructure has been developed to enhance both SERS and the detection of fluorescence through plasmon amplification. The strong “antenna” and “hot spot” effects that the 3D-RCW structure demonstrates are essential for producing SERS and plasmon-enhanced fluorescence (PEF) effects. Their studies show that the RCW nanochip, intended for both emission and Raman-enhanced detection, analyzes extremely tiny sample quantities. With LODs ranging from 5 µM to 0.05 µM in just 20 µL of the sample, they were able to analyze and describe the SERS and PEF of pesticides such as thiram, carbaryl, paraquat, and fipronil. Notably, 3D plasmon-enhanced platform increased the fluorescence of weak emitters (pesticides) by over 1,000 times using SPR excitation in addition to collecting SERS signals from pesticides. Fluorescence biosensors’ capabilities might be increased by this development (Table 2) [[112], [113], [114], [115], [116], [117]].

**Table 2:** Applications of SERS in different areas.

Substrate	Analyte	Sample or sample matrix	LOD	Refs.
Silver nanoparticles	Carbendazim	Spiked orange juice & kale leaves	<2 mg/L	[112]
Gold/Zno nano urchins	Thiram	Analyte on SERS surface	10 pM	[113]
Ferro chocolate like copper oxide @silver microspheres	Thiram	Apple peels	0.018 mg/L	[114]
Gold nanoparticles	Thiram, phorate, benthiocarb	Rice, vegetables & fruits	10^−8^ g/mL for Thiram & 10^−6^ g/mL for phorate	[115]
Gold nanoparticles	Chlorpyrifos	Tap water	–	[116]
Gold nanoparticles	Thiram, carbaryl, paraquat & fipronil	Analyte on SERS substrate	5–0.05 μM in 20 μL	[117]

–: no data; LOD: limit of detection; SERS: surface-enhanced Raman spectroscopy.

Ibáñez et al. [118] described identification of cancer using particles that are encoded with SERS. With the potential to significantly outweigh the considerable limitations associated with conventional techniques, optical biosensors have emerged as a very promising field within nanomedicine. Possibilities for using SERS-encoded particles in the operating room are made possible by the employment of SERS-encoded particles as contrast agents and improvements in making Raman systems smaller. To thoroughly remove any leftover tumors at the edges, this can provide surgeons with real-time guidance during tumor removal surgery. The two unique strategies that are being considered include applying SERS-encoded particles topically and injecting them systemically. Using SERS-encoded particles, SERS sensing has developed into a reliable analytical technique with several applications in cancer research. These applications include tumor cell analysis, immunohistochemistry (IHC), surgical resection guidance, and solid tumor localization for bioimaging and staging. Their primary emphasis was directed towards the most recent breakthroughs in optical biosensors that utilize SERS-encoded particles. These advancements were particularly focused on characterizing individual tumor cells at the molecular level and studying tissues through techniques like IHC. Moreover, they explored the potential of these techniques in guiding surgical procedures and even in conducting bioimaging within living organisms.

Huang et al. [119] proposed the use of SERS for identification of fungi, bacteria, and viruses. The groundbreaking finding of employing SERS for detecting fungal samples and assigning certain Raman peaks to their constituents was discovered in 1995 by Edwards and colleagues [120]. Xia et al. [121] later confirmed that the lipids in the cell membrane and the polysaccharides, such as chitin and amylopectin, in the fungal cell wall were the main sources of the Raman signals. Raman spectroscopy is a method for examining scattered light from a substance. It’s crucial to keep in mind that the dispersed signal’s strength is extremely low, making it difficult to obtain reliable conclusions. SERS is unsuitable for researching complex live organisms because of these drawbacks. SERS offers exceptional photobleaching resistance, sensitivity in detection, and the ease of non-destructive in-situ testing. SERS may be used effectively to identify and classify fungi. Edwards et al. [122] carefully examined the fungi’s constituent parts before comparing the resultant spectra to reference Raman spectra that are known to exist in macrofungi, which contain compounds like saccharides. Various methods, both in synthetic preparation and microelectromechanical system processing, can be employed to create SERS substrates that exhibit consistent and reliable performance. These methods include chemical reduction, magnetron sputtering, electron beam lithography, etc. Mabbott et al. effectively distinguished and categorized various fungal species, including Candida albicans, Candida glabrate, Candida cruises, and Aspergillus, using silver hydroxylamine NPs modified with single-stranded DNA as SERS markers, combined with principal component analysis. A silver–gold bimetallic composite was formed when Sivanesan et al. employed potentiostatic electrodeposition to install a thin gold layer over a rough nano-silver substrate. Sivanesan et al. conducted SERS spectroscopy to identify three different genera of fungi: Trichophyton, Microsodomia, and tinea epidermis. Gussem et al. [123] employed the chemical reduction method to synthesize AgNPs and successfully utilized SERS technology, supplemented by principal component analysis and linear discriminant methods, to identify Aspergillus fumigatus. Dina et al. [124] developed aptamers for a SERS sensor chip. They employed this chip, along with SERS label preparation, complementary DNA hybridization, and other intricate processes, to successfully detect mycotoxins, specifically aflatoxin B1. The study of bacteria, fungi, and cells has tremendous potential and benefits when paired with microfluidic chip technology and SERS analysis. To do this, a SERS analysis component must be added to a microfluidic chip. It makes it possible to introduce biochemical samples that contain fungus into the microfluidic chip’s integrated nanostructured SERS detecting zone [125].

Boardman et al. [126] evaluated the use of a signal-optimized, label-free sensor based on hybrid Au-Ag NPs for simultaneous detection of benzimidazole fungicides in food samples. Galvanic displacement-free deposition was used to build a label-free anisotropic bimetallic hollow gold-silver nano star (Au/Ag NS) SERS substrate with plenty of Raman hotspots [127]. Using a particular peak at 1,224 cm^−1^ (carbendazim) and 778 cm^−1^ (thiabendazole), the calibration results for solo and combined analytes were examined, and the findings showed minor changes. Recovery rates in rice and water samples ranged from 91.54% to 98.26%, according to accuracy and precision analyses. In the end, the HPLC-based validation results were judged to be adequate (p > 0.05). The LOD found for both CBZ and TBZ were 4.28 × 10^−4^ and 6.04 × 10^−4^ μg/mL respectively. The calculated limit of quantitation (LOQ) were 5.09 × 10^−4^ and 7.92 × 10^−4^ μg/g for CBZ and TBZ, respectively. At 1,224 and 778 cm^−1^ of each solution of CBZ, the calibration plot of various concentrations vs. SERS signal intensity yielded r^2^ values of 0.9994 and 0.9996, respectively. The calibration plot of different concentrations vs SERS signal intensity at 1,224 and 778 cm^−1^ for individual solutions of TBZ are 0.9991 and 0.9989, respectively. The main objective of the project is to develop a simple, label-free, and highly sensitive SERS sensor by combining HAu/Ag NS with Discrete Fourier Transform (DFT) and Solid Phase Extraction (SPE). Various concentrations of concentrated AgNO_3_ solution were used for optimizing the SERS signal, and it was discovered that 9 mL of the solution produced the maximum EF, which was 1.21 × 10^8^. The detection of both CBZ and TBZ was then carried out using this improved EF. Both in separate solutions and when the two substances were combined, a linear connection between their respective concentrations and the SERS signal intensity was seen over concentration ranges for CBZ and TBZ extending from 0.001 to 100 g/mL. The simultaneous determination of both drugs in rice and water could be done using the Au/Ag NS SERS sensor linked SPE to ensure the quality and safety of food. Further research is needed to use the suggested approach in different food matrices[128].

Wang et al. [129] studied unlabeled differentiation of glioma brain tumors at various stages using SERS. A quick SERS technique was developed using AgNPs arranged on silver nanorods (AgNPs@AgNR) as substrates for distinguishing gliomas. The AgNPs@AgNR substrates exhibited exceptional SERS capabilities, boasting an EF of up to 1.37 × 10^9^ in comparison to silver nanorod substrates with SERS-active AgNPs. They successfully differentiated between healthy brain tissue and gliomas of varying grades. They told us whether tissue was from a healthy region or a glioma based on the considerable differences in the spectral data we acquired from the tissue samples. The difference between the SERS spectra of healthy brain tissue and various-grade gliomas is particularly remarkable for having a decreased ratio of two distinguishing peaks at 653 and 724 cm^−1^. They could separate normal brain tissue Grade II gliomas, as well as between Grade III and Grade IV gliomas, using three-dimensional PCA analysis. These preliminary findings suggested that SERS spectra produced from AgNPs@AgNR substrates are ideal for rapid detection due to specimen preparation procedure and the speedy collection of spectral data. They increased their sample size to reduce the possibility of both inaccurate positive and negative outcomes, enhancing the precision and dependability of their study. This strategy is a useful technique for making a clinical diagnosis in future prospects.

The advantages and disadvantages are provided below in Table 3, Table 4 for general considerations, and specific applications may have unique requirements that could influence the suitability of LSPR and SERS, respectively.

**Table 3:** The basic advantages and disadvantages of different types of localized surface plasmon resonance (LSPR).

Type of LSPR	Advantages	Disadvantages
Nanoparticles (NPs)	Easy to synthesize and functionalize	Limited tunability of LSPR peak wavelengths
	High surface area for enhanced sensing	Sensitivity to NP size, shape, and aggregation state
	Can be used in solution and on surfaces	Batch-to-batch variability in NP synthesis
Nano stars/nanoflowers	Highly tunable LSPR peaks and sharpness	Elaborate synthesis methods and reaction conditions
	Excellent signal enhancement	Difficulties in achieving uniform size and shape
	Plasmonic coupling for enhanced effects	Prone to aggregation issues
Nanorods/nanostars	Tunable LSPR peaks with aspect ratio	Complex synthesis methods and control parameters
	Strong plasmonic coupling effects	Aggregation tendency leading to signal variability
	Enhanced sensitivity due to anisotropy	–
Nanorings/nanodisks	High sensitivity and improved signal	Complex fabrication methods and equipment requirements
	Enhanced electric field confinement	Limited scalability for large-scale applications
	Strong dependence on ring/disk geometry	
Nanoshells/nanocages	Versatile tunability of LSPR peaks	Limited enhancement in comparison to other shapes
	Good biocompatibility for biomedical apps	Requires precise control over shell thickness
	Enhanced stability compared to NPs	Difficulties in achieving uniform shapes and sizes

–: no data.

**Table 4:** The fundamental pros and cons of various surface-enhanced Raman scattering (SERS) types.

SERS technique	Advantages	Disadvantages
Substrate based SERS	Better reproducibility	Time-consuming fabrication process
	Enhanced stability	Limited enhancement factors compared to colloidal SERS
	Suitable for various sample geometries	Requires careful substrate preparation
Tip-enhanced SERS	Extremely high enhancement factors	Highly specialized equipment and probes
	Nanometer-scale spatial resolution	Limited field of view, small sampling area
	Potential for single-molecule detection	Challenging tip fabrication and alignment
Colloidal SERS	Easy to prepare	Lack of reproducibility
	High enhancement factors	Limited stability of colloidal solutions
	Suitable for lab-scale experiments	Difficult to control nanoparticle size

The sensitivity to double plasmonic peak shifts has become a prominent subject in contemporary research. For instance, there is a current focus on advanced LSPR-PCF sensors known for their high sensitivity, enabling the detection of a wide array of analytes [130]. For effective strategies for improving photocatalytic activity nowadays, LSPR technologies are widely used. Wang et al. [131] loaded varying amounts of silver quantum dots onto extremely thin carbon nitride sheets (Ag/UCN) to create a Schottky junction. The Schottky junction with a 1:1 ratio of silver to UCN shows outstanding performance in breaking down ofloxacin, achieving a removal rate of 95.2%. Moreover, it maintains high stability over four consecutive rounds of photocatalysis. Nowadays, graphene-based substances are being used in SERS for their good surface area, good chemical stability, and tunable electrical properties [132]. Semiconductor substances in SERS are popular because of their compatibility with electronic devices. The concept of hybrid SERS substrates is novel, characterized by heightened sensitivity, customization for specific applications, and the enhancement of signal strength [133]. Table 4 explains different types of SERS with their advantages and disadvantages in the modern era.

## 8 Conclusions

The proposed review is intended to provide a thorough overview of the latest advancements in optical biosensors using LSPR and SERS technologies. This comprehensive description encompasses their fundamental spectroscopic characteristics, underlying principles, and operational mechanisms. Furthermore, it also delivers the significant applications of these biosensors in the biomedical sciences, environmental monitoring, food safety and clinical analysis. This review also anticipates the future demands and growth trends in biosensing field. It systematically and comprehensively assesses the progression of plasmonic analyte sensors, drawing insights from research and review papers published over the past five years. NPs are useful for biosensor applications because they are essential for enhancing SERS and LSPR signals. It aims to offer valuable information to researchers. A Biosensor chip has been created, featuring a high-density deposition of AuNPs under varying ligand concentrations and reaction durations on the substrate. This development has led to the establishment of an LSPR chip detection platform, opening up innovative possibilities for portable sensor technology. A typical explanation of sensogram of changing RI due to specific binding, δ dielectric-evanescent field of the dielectric, δ metal-evanescent field in the metal, δ SPP is properly discussed. This review confirms and elucidates the advancements in ongoing research as well as the significance of SERS over the LSPR technology. This study focuses on the applications of optical biosensors in the detection of different disease biomarkers. LSPR and SERS both play a vital role in detection of various types of cancers, viral diseases, fungal diseases, bacterial infectious diseases, and cardiac disease biomarker detections, as well as environmental monitoring, food safety, refining of impurities from water samples, etc. Standard analytical methods can be improved by using biomarkers for detection and prognosis of cancer, and HIV-infected patients by LSPR and SERS. AgNP substrates provide versatile applications for cancer therapy and cancer management. In the tabular form and the data acquired regarding LOD, LOQ, target analyte, and substrate all are discussed with proper biomarker identification. In future progress this biosensor system will come with great turning point of capability of simultaneous multi-analyte monitoring with the good stability, good precision, and tunable specificity. The prospective development of biomarker detection through LSPR and SERS methods shows significant promise in the clinical realm. These advanced techniques have the potential to transform diagnostic procedures, offering swift, precise and sensitive detection of biomarkers linked to various diseases. Early disease identification, simultaneous detection of multiple biomarkers, point-of-care testing, quantitative analysis, and research on the biocompatibility and stability of these techniques represent practical future avenues for biosensors. In essence, the future of biomarker detection using LSPR and SERS methods in clinical applications holds immense potential. Ongoing research, innovation, and collaboration across diverse fields are vital to surmounting challenges and fully realizing the clinical benefits of these sophisticated diagnostic technologies. The use of LSPR/SERS enables us to get fast, reliable and accurate measurements, high sensitivity and outstanding results in all the analyte concentration ranges. Despite many promising advancements, the understanding is still limited, and translation of these two biosensors to clinical applications remains challenging.

## **CRediT authorship contribution statement**

**Bibhu Prasad Nanda:** Writing – original draft. **Priyanka Rani:** Writing – original draft. **Priyanka Paul:** Investigation. **Aman:** Resources. **Subrahmanya S. Ganti:** Validation. **Rohit Bhatia:** Data curation, Validation.

## Declaration of competing interest

The authors declare that there are no conflicts of interest.
